# Rationale and design of the Novel Uses of adaptive Designs to Guide provider Engagement in Electronic Health Records (NUDGE-EHR) pragmatic adaptive randomized trial: a trial protocol

**DOI:** 10.1186/s13012-020-01078-9

**Published:** 2021-01-07

**Authors:** Julie C. Lauffenburger, Thomas Isaac, Lorenzo Trippa, Punam Keller, Ted Robertson, Robert J. Glynn, Thomas D. Sequist, Dae H. Kim, Constance P. Fontanet, Edward W. B. Castonguay, Nancy Haff, Renee A. Barlev, Mufaddal Mahesri, Chandrashekar Gopalakrishnan, Niteesh K. Choudhry

**Affiliations:** 1 Division of Pharmacoepidemiology and Pharmacoeconomics, Department of Medicine, Brigham and Women’s Hospital and Harvard Medical School, 1620 Tremont Street, Suite 3030, Boston, MA 02120 USA; 2 Center for Healthcare Delivery Sciences (C4HDS), Department of Medicine, Brigham and Women’s Hospital and Harvard Medical School, 1620 Tremont Street, Suite 3030, Boston, MA 02120 USA; 3 Atrius Health, Newton, MA USA; 4 Dana-Farber Cancer Institute, Department of Biostatistics and Computational Biology, Harvard T.H. Chan School of Public Health, Boston, MA USA; 5 Tuck School of Business, Dartmouth College, Hanover, NH USA; 6 Ideas42, New York, NY USA; 7 Division of General Internal Medicine and Department of Health Care Policy, Brigham and Women’s Hospital and Harvard Medical School, Boston, MA USA; 8 Marcus Institute for Aging Research, Hebrew SeniorLife, Boston, MA USA

**Keywords:** Deprescribing, Older adults, Decision support, Pragmatic trial, Prescribing, Adaptive trial

## Abstract

**Background:**

The prescribing of high-risk medications to older adults remains extremely common and results in potentially avoidable health consequences. Efforts to reduce prescribing have had limited success, in part because they have been sub-optimally timed, poorly designed, or not provided actionable information. Electronic health record (EHR)-based tools are commonly used but have had limited application in facilitating deprescribing in older adults. The objective is to determine whether designing EHR tools using behavioral science principles reduces inappropriate prescribing and clinical outcomes in older adults.

**Methods:**

The Novel Uses of Designs to Guide provider Engagement in Electronic Health Records (NUDGE-EHR) project uses a two-stage, 16-arm adaptive randomized pragmatic trial with a “pick-the-winner” design to identify the most effective of many potential EHR tools among primary care providers and their patients ≥ 65 years chronically using benzodiazepines, sedative hypnotic (“Z-drugs”), or anticholinergics in a large integrated delivery system. In stage 1, we randomized providers and their patients to usual care (*n* = 81 providers) or one of 15 EHR tools (*n* = 8 providers per arm) designed using behavioral principles including salience, choice architecture, or defaulting. After 6 months of follow-up, we will rank order the arms based upon their impact on the trial’s primary outcome (for both stages): reduction in inappropriate prescribing (via discontinuation or tapering). In stage 2, we will randomize (a) stage 1 usual care providers in a 1:1 ratio to one of the up to 5 most promising stage 1 interventions or continue usual care and (b) stage 1 providers in the unselected arms in a 1:1 ratio to one of the 5 most promising interventions or usual care. Secondary and tertiary outcomes include quantities of medication prescribed and utilized and clinically significant adverse outcomes.

**Discussion:**

Stage 1 launched in October 2020. We plan to complete stage 2 follow-up in December 2021. These results will advance understanding about how behavioral science can optimize EHR decision support to improve prescribing and health outcomes. Adaptive trials have rarely been used in implementation science, so these findings also provide insight into how trials in this field could be more efficiently conducted.

**Trial registration:**

Clinicaltrials.gov (NCT04284553, registered: February 26, 2020)

**Supplementary Information:**

The online version contains supplementary material available at 10.1186/s13012-020-01078-9.

Contributions to the literature

- Novel Uses of adaptive Designs to Guide provider Engagement in Electronic Health Records (NUDGE-EHR) is a 16-arm adaptive randomized pragmatic trial to identify the most effective of numerous electronic health record tools for prescribing.
- Adaptive randomized trials, like the NUDGE-EHR trial, have rarely been used in implementation science, largely due to a limited understanding of practicalities conducting these trials.
- Regardless of the outcomes of NUDGE-EHR itself, our approach offers important lessons for the conduct of trials in implementation science, especially because methods to identify how to best deliver evidence-based interventions and more efficiently generate evidence are central to implementation science.

## Background

Prescribing of potentially unsafe medications for older adults remains extremely common [1–3]. More than 20% of older adults are chronically using at least one of these medications [3–5]. Chronic use can result in adverse health consequences such as an increased risk of hospitalizations, falls, and fractures [3, 6]. For instance, benzodiazepines and sedative hypnotics are thought to increase the 1-year risk of falling by 30% in older adults, even among patients who have been using them [7, 8]. Despite strong clinical guidelines recommending reductions in their use, a gap persists in how to achieve deprescribing of high-risk medications in clinical practice.

Several prior studies have evaluated strategies to de-implement the prescribing of high-risk medications to older adults [4, 9–11]. Specific approaches have included in-person patient education, pharmacist medication or drug utilization reviews, clinician-facing education, or referral to specialist care [11–13]. The vast majority of these have been complex and multi-faceted, and the timing of intervention provision has also varied; some were delivered at specific points in care which may be too late, such as hospital or nursing home admission [14]. Collectively, these interventions have been only modestly effective, perhaps in part due to issues of design or implementation, and substantial resources would be required to sustain their use.

Computerized clinical decision support tools in electronic health record (EHR) systems are a widely scalable strategy to influence physician behavior and have demonstrated effectiveness in implementation science interventions to increase the uptake of preventive health screenings, test ordering, and prescribing guideline-indicated medications [9, 15, 16]. Several prior studies have evaluated the use of decision support to facilitate the deprescribing of high-risk medications to older adults and have found inconsistent results [17–21]. The lack of effectiveness of these EHR interventions may have resulted from aspects of their designs, such as their not presenting clinically actionable information to overcome true barriers to de-prescribing like clinical inertia, patient pressure, or limited access to alternative treatments, or the decision support being insufficiently focused on workflow and cognitive heuristics, such as providing large blocks of text to communicate risks [22–24]. Their usability and likelihood of producing success moving forward could also be hampered further by barriers recognized by implementation frameworks, like the representativeness of participating providers and the timing and cost of the interventions [25, 26].

Accordingly, the effectiveness of EHR decision support tools for reducing the prescribing of high-risk medications may be improved by incorporating design principles of behavioral sciences, such as framing, pre-commitment/consistency, or boostering, which have demonstrated impact on individual behavior in other contexts [21, 27–33]. For example, providing information in terms of risks rather than benefits can make patients more willing to fill a prescribed medication [27, 34]. Similarly, using “defaults” within EHRs can increase generic medication prescribing and ordering of recommended laboratory tests [32].

To fill this knowledge gap, we initiated Novel Uses of adaptive Designs to Guide provider Engagement in Electronic Health Records (NUDGE-EHR) trial. Because there are numerous ways in which EHR tools that incorporate behavioral science principles could be designed and because a direct comparison of approaches is typically difficult to accomplish with parallel-group trials [35, 36], thus, NUDGE-EHR uses an adaptive design, a methodology that has rarely been used in implementation research [36]. Using this design will help identify the best way to deliver deprescribing tools within an EHR and will provide guidance on which specific components of these tools are most effective at changing prescribing, which will inform implementation in other settings. Regardless of study findings, NUDGE-EHR could be used as a blueprint about how to consider and overcome the practicalities of conducting adaptive trials in implementation science.

## Methods/design

### Overall study design

**Fig. 1:**
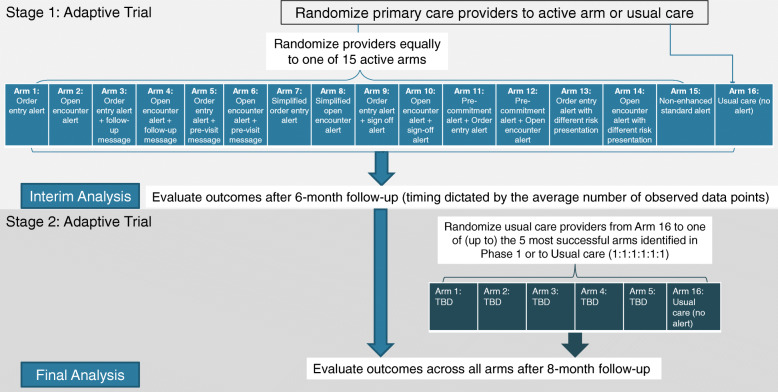
Overview of the adaptive trial stages

NUDGE-EHR is a two-stage adaptive randomized pragmatic trial with a “pick the winner” design that seeks to identify the optimal EHR tool for reducing the use of benzodiazepines, sedative hypnotics (“Z-drugs”), and anticholinergics among older adults (Fig. 1). In stage 1, primary care providers (PCPs) at a large integrated delivery network have been randomized to one of 15 active intervention arms or usual care. For stage 2, we will randomize (a) stage 1 usual care providers to one of up to the top 5 promising stage 1 treatment arms or to continue usual care and (b) stage 1 providers in the unselected arms (e.g., ranked lower than 5) will be randomly assigned to one of up to the top 5 promising arms or usual care.

The study is funded by the NIH National Institute on Aging. The trial was approved by the Mass General Brigham institutional review board who waived informed consent for all subjects. The study is registered with clinicaltrials.gov (NCT04284553, first registered February 26, 2020) and overseen by a Data and Safety Monitoring Board. The authors are solely responsible for the design and conduct of this study and drafting and editing of the paper and its final contents.

The trial was designed using Pragmatic Explanatory Continuum Indicator Summary (PRECIS-2) trial guidance and reported using SPIRIT reporting guidelines (full protocol in Supplement 1).

### Study setting and subjects

NUDGE-EHR is being conducted at Atrius Health, a delivery network in Massachusetts that uses the Epic® EHR system, the platform used by > 35% of US ambulatory practices and 60% of large hospitals [37]. Atrius employs approximately 220 PCPs at 31 practices who care for approximately 745,000 patients. Eligible study participants include these PCPs (including physicians and PCP-designated nurse practitioners and physician assistants) and their patients.

Providers are eligible if they prescribed a high-risk medication to ≥ 1 older adult in the 180 days before stage 1 assignment. Eligible high-risk medications include benzodiazepines, Z-drugs (e.g., zolpidem), and anticholinergics and were chosen based on clinical guidelines like Choosing Wisely and Beers Criteria that recommend reductions in use and they continue to be over-prescribed [38–40]. For stage 1, eligible patients of these providers are those aged ≥ 65 years and who have been prescribed ≥ 90 pills of benzodiazepine or Z-drug in the last 180 days, which most guidelines consider “chronic” use [40]. For stage 2, eligibility will be identical to stage 1 except that patients (and their providers) will also be included in secondary analyses if they had been prescribed ≥ 90 pills of eligible anticholinergics in the last 180 days. We chose to include just benzodiazepines and Z-drugs in stage 1 as these medications have similar prescribing patterns and likely have less variation with improved statistical power. For stage 2, we also included anticholinergics to ensure generalizability to other classes.

### Interventions

Our intervention design was based upon a thorough review of peer-reviewed literature and two focus groups with PCPs intended to understand barriers to and facilitators of appropriate prescribing. Based on their effectiveness in other settings, applicability to older adults, and ability to be adapted to EHRs [30, 41–43], we selected nine behavioral principles for testing: salience, default bias, social accountability, timing of tools (e.g., an aspect of choice architecture), boostering, “cold-state” priming, simplification, pre-commitment/consistency, and framing (Table 1) [28, 33, 42, 44, 45]. Using these principles, we co-designed and iteratively tested the EHR tools with our multidisciplinary team in the healthcare system.

**Table 1:** Behavioral principles tested in the electronic health record tools across study arms

Behavioral principle	Definition in context of medication prescribing	Modification made to electronic health record tool to incorporate the principle	Trial arm(s) with this modification
Salience	Noticeability or prominence of drug risk information	Presenting information about drug risks in ways that makes the information as impactful as possible	1–14
Default bias	Pre-set course of action that leverages providers’ tendency to do status quo	Defaulting the options to (1) discontinuing the medication and (2) opening an order set that contains dose tapers, alternatives, and customized patient instructions	1–14
Social accountability	Willingness to accept responsibility for prescribing action	Requiring providers to select “I accept the drug’s risks” or write a free-text response if they decide not to discontinue the medication or order a taper or alternative	1–14
Timing of information (an aspect of choice architecture)	Organization of the context and timing in which providers make choices to influence decision-making	Modifying the timing of the tool to occur at different times in provider workflow (i.e., when ordering a medication, opening an encounter record, or approving a medication refilled by clinical staff)	*-At ordering:* 1, 3, 5, 7, 9, 11, 13, 15 *-At encounter opening*: 2, 4, 6, 8, 10, 12, 14 *-At refill approval:* 9, 10
Boostering	Renewal of the effect of a prior intervention	Providing an option for reminder message 4 weeks after a patient visit	3, 4
“Cold-state” priming	Pre-exposure to information affects subsequent prescribing	Triggering an informational message 2 days before a scheduled visit with an eligible patient	5, 6
Simplification	Simplification of risk information may make it more understandable	Simplifying the medication risks and recommended action language in the tool	7, 8
Pre-commitment/consistency	Support of future decision to deprescribe by first asking providers to commit to an easier action	Prompting providers to discuss medication risks on their first visit (using a patient hand-out in the EHR) before prompting providers to stop medication or order a dose taper when the patients return for follow-up	11, 12
Framing	Framing of risks in terms of clinical guidelines	Framing of risks in terms of clinical guidelines and published evidence	13, 14

Abbreviation: *EHR* Electronic health record

**Fig. 2:**
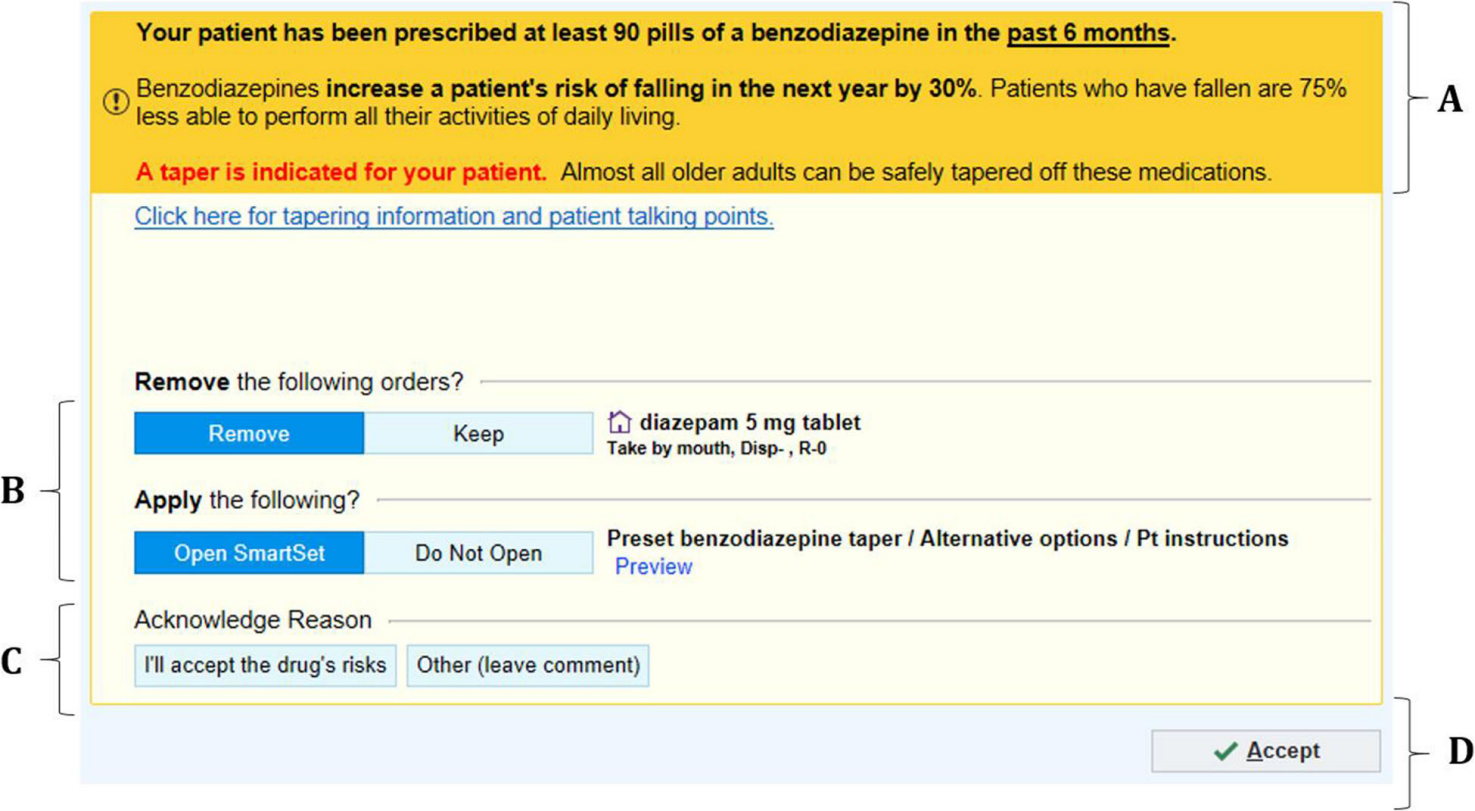
Enhanced electronic health record tool modified with behavioral principles that triggers when ordering one of the high-risk medications. **a** Salience: Presenting information about risks impactfully. **b** Defaults: Defaulting options to (1) discontinuing the order and (2) opening an order set containing dose tapers, alternatives, and patient instructions. **c** Social accountability: Requiring providers to select either “I accept the drug’s risks” or write a free-test response if they do not discontinue the medication or order a taper. **d** Choice architecture: Modifying timing of the tool to occur at different times in provider workflow

**Fig. 3:**
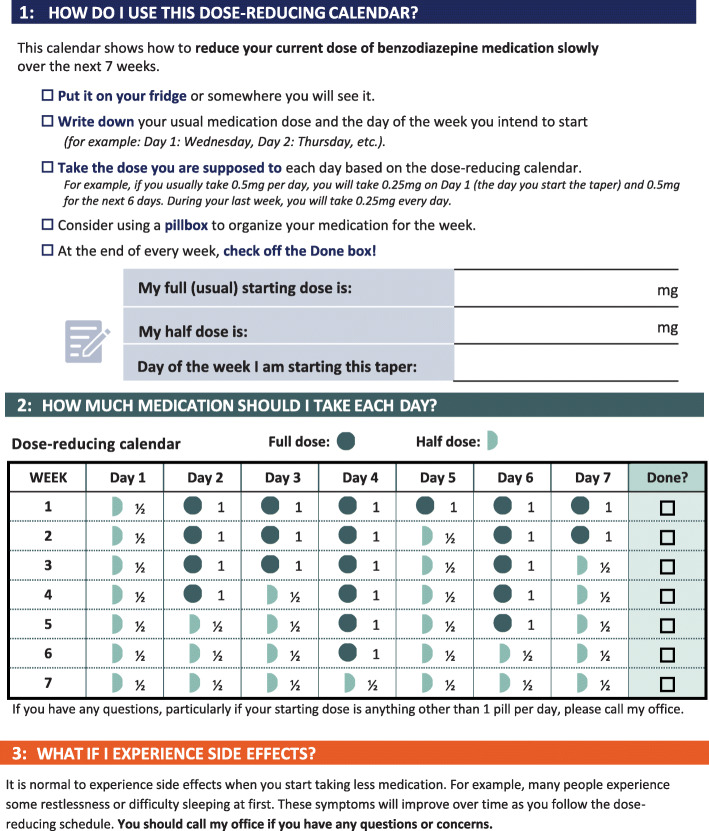
Customized patient instructions for medication tapering algorithms. The instructions for the first prescription for a dose reduction of a once-daily benzodiazepine medication is shown

Arms 1 through 14 of stage 1 involve EHR tools that incorporate the selected behavioral science principles (Fig. 2). The central component of each intervention is an enhanced EHR alert (Best Practice Advisory [BPA] in Epic®). This alert provides information about risks of continued medication use and contains an embedded hyperlink to tips to help providers discuss medication discontinuation with their patients (Supplement 2, eFigure 1). When the alert triggers, it defaults to an order set (a SmartSet in Epic®) that provides alternative treatments, templated patient instructions, and relevant referrals, such as to behavioral health. For the benzodiazepines and Z-drug alerts, the order set also includes tapering algorithms to limit risks of withdrawal symptoms. These algorithms are customized to the specific drug, dose, and frequency that the patient is taking along with pre-filled directions to pharmacies dispensing the taper (Supplement 2, eFigure 2) and customizable and printable instructions for patients about how to gradually taper off these drugs (Fig. 3). The order set also allows providers to order alternative medications, refer patients to a behavioral health specialist, and share instructions with patients about how to make lifestyle modifications to improve symptoms.

Arms 1 and 2 are “base” interventions in which the alert either fires when the provider orders a medication (arm 1) or opens an encounter (arm 2) for eligible patients during in-person or telehealth visits, which require addressing before completing the intended action (Supplement 2, eFigure 3). Additional principles are incorporated in arms 3–14. In arms 3 and 4, providers have the option of receiving a follow-up “booster” message (Supplement 2, eFigure 4). Arms 5 and 6 incorporate “cold state” priming, where providers receive a message in the EHR inbox 2 days before a scheduled visit with an eligible patient. Arms 7 and 8 involve simplified language (Supplement 2, eFigure 5), whereas arms 9 and 10 test an additional alert that occurs at the time of approving a medication when it is reordered or refilled by support staff such as a medical assistant (Supplement 2, eFigure 6). Arms 11 and 12 incorporate pre-commitment, in which providers are prompted to first have a discussion with their patient about medication risks and then, at a subsequent visit, are prompted to actively deprescribe the relevant medication (Supplement 2, eFigure 7). Arms 13 and 14 test framing risks based on guidelines and evidence instead of just presenting the risks quantitatively (Supplement 2, eFigure 8).

Conversely, arm 15 is a basic EHR alert meant to mimic clinical decision support that is currently most commonly given to providers, i.e., without behavioral science (Supplement 2, eFigure 9). Physicians randomized to usual care (arm 16) receive no new EHR alert. Presently, a system-generated informational alert fires for all providers upon ordering a benzodiazepine; this will fire across all arms non-differentially.

If patients are eligible for multiple classes of interest (e.g., benzodiazepines and Z-drugs), the EHR tools will appear simultaneously. To supplement the two focus groups, we also incorporated feedback from the Internal Medicine Design team and clinical pharmacists at Atrius. Finally, to fully ensure user-centered design, we conducted pilot testing with several providers not in the trial to assess usability, feasibility, and appropriate firing of the tools following Agency for Healthcare Research and Quality principles [46].

### Stage 1

#### Randomization

In stage 1, we stratified the 201 eligible PCPs into 4 blocks (with 50 PCPs in 3 of the blocks and 51 PCPs in the fourth) based on clinical practice size (i.e., number of providers) and baseline rates of high-risk prescribing (i.e., number of eligible patients). Within these blocks, 30 PCPs were randomly allocated using a random number generator to one of 15 active arms, and 20 PCPs were allocated to usual care (in the 4th block, the extra PCP was assigned to usual care). In this way, 120 PCPs were assigned to an active intervention (*n* = 8 per intervention), and 81 PCPs were assigned to usual care. Providers were randomized using data from March 1 to August 31, 2020, and randomization was performed in September 2020; stage 1 began in October 2020.

#### Outcomes

The trial outcomes are shown in Table 2 and will be assessed using routinely collected structured EHR data and/or administrative claims data on the patient level. The primary outcome is a “reduction” in inappropriate prescribing defined as either discontinuation of one of the medication classes of interest or ordering of a medication taper. If either action is taken by the provider for a specific patient in the 6-month follow-up, we will classify the patient as having had a reduction in inappropriate prescribing, even if there is an unexpected later escalation. If a patient is eligible for > 1 therapeutic class (e.g., a benzodiazepine and a Z-drug), we will consider a patient as having met the primary outcome if there was a reduction in any of their eligible medications. This primary outcome was chosen because it can be rapidly measured to facilitate the adaptive trial. In secondary analyses, we will analyze outcomes stratified by the patient’s number of eligible therapeutic classes (i.e., one, two, or three).

**Table 2:** Trial outcomes

Type	Outcome	Data source	Definition	When outcome is analyzed
*Primary*	Reduction in inappropriate prescribing	EHR	Composite of (1) discontinuation of high-risk medications or (2) ordering a gradual dose taper	End of stages 1 and 2
*Secondary*	Quantity of prescribing	EHR	Number of milligram equivalents of high-risk medications prescribed to patients	End of stage 2
*Tertiary*	Quantity of medication dispensed	Administrative claims	Number of milligram equivalents of high-risk medications filled by patients	End of stage 2
	Adverse events	Administrative claims	Occurrence of clinically significant adverse drug events, such as all-cause hospitalizations, falls, and fractures	End of stage 2

#### Analysis plan

Six months after stage 1 launch, we will use multivariable regression to determine which of the behavioral components in the EHR tools (e.g., boostering effects) is associated with a reduction in inappropriate prescribing (primary outcome) among eligible patients who presented to care. The behavioral components are shown as factors for the models in Supplement 2, eTable 1. In specific, we will adjust for physician-level clustering and multiple patient observations per physician using a generalized linear mixed model for binary outcomes. Based upon this analysis, the 15 active arms will be ranked based on their observed effect estimates compared with usual care. Sample size estimates are described in stage 2. We will only present the study results at the end of stage 2.

### Stage 2

#### Randomization

After the stage 1 analysis, we will randomize the 201 stage 1 providers as follows: (a) stage 1 usual care providers will be randomized to one of up to the top 5 most promising treatment arms or to continue to receive usual care (1:1:1:1:1:1) (Fig. 1), (b) stage 1 providers who were previously in unselected treatment arms (e.g., ranked lower than 5) will be randomized to one of up to the top 5 most promising arms or to usual care (1:1:1:1:1:1), and (c) stage 1 providers who were previously in the most promising arms will be randomly assigned to continue to receive their original treatment assignments or to usual care to test holdover/persistency effects (1:1). If only 1 to 5 arms are ranked, we will choose those for testing in stage 2 and randomize PCPs in equal proportions. If none of the arms are promising versus usual care, we will combine intervention components within the arms to enhance effectiveness.

In stage 2, as secondary analyses, we will also include and randomize any additional providers who prescribed ≥ 1 eligible anticholinergic of ≥ 90 days to an older adult in the prior 180 days and include these in the analysis. Based on preliminary data, we expect an additional 10–15 providers. We will also stratify based on clinic size and baseline rates of prescribing in stage 2. Follow-up will last approximately 8 months but will be based on the average number of observations.

#### Outcomes

As in stage 1, the primary outcome will be a reduction in inappropriate prescribing defined by discontinuation of high-risk medications or ordering a gradual dose taper. The measurement approach will be the same as for stage 1. In secondary analyses, we will include anticholinergics; the primary outcome for this definition will be a reduction in inappropriate prescribing defined by discontinuation of high-risk medications (benzodiazepines, Z-drugs, or anticholinergics) or ordering a gradual dose taper (benzodiazepines or Z-drugs).

At the end of stage 2, we will also measure other prescribing and clinical outcomes from the EHR including the cumulative number of milligram equivalents of high-risk medications prescribed to patients (secondary outcome). Because claims data are available for a subset of patients whose insurance is in risk-bearing contracts with Atrius, we will also measure tertiary outcomes including the quantity of high-risk medications dispensed in follow-up, measured in pharmacy claims, and the occurrence of clinically significant adverse drug events, specifically diagnoses of sedation, cognitive impairment, and falls or fractures, and all-cause hospitalizations, measured in medical claims data.

We will also measure implementation outcomes informed by the RE-AIM and CFIR implementation frameworks to provide insight into its implementation and scalability to other healthcare settings [25, 47]. These outcomes will include characteristics of providers evaluated in the trial compared with other Atrius providers, percentage and frequency of decision support firing as intended, frequency of using the SmartSet order set, and feedback from clinics and providers about the intervention about acceptability and sustainability after completion of the trial.

#### Analytic plan

We will conduct analyses of the primary outcome using generalized linear mixed model for binary outcomes to determine whether any of the intervention arms are more effective versus usual care. For secondary and tertiary outcomes, we will also use generalized linear mixed models. Primary analyses will be unadjusted; however, if there are strong patient-level predictors not balanced by stratified randomization, we will adjust for these. We will not include multiplicity adjustments in our statistical plan.

#### Sample size

Our null hypothesis is that rates of provider prescribing (defined as a reduction in prescribing of high-risk medication) in any of the active intervention arms will be no different than usual care. We determined our sample size to have 80% power assuming that the baseline rate of the composite outcome among usual care physicians is 5% (i.e., that 5% of patients would have a medication discontinued or a taper ordered in follow-up independent of the intervention) and that the effective interventions will increase the relative rate of deprescribing for patients by 10% (i.e., an odds ratio of 1.10) with type I error = 0.05 and correlation = 0.3 [11, 18, 48]. We assumed an average cluster size of 20 patients per provider based on baseline data.

### Limitations

There are several potential limitations. These trials are being conducted in one health system. The results may also not be applicable to initial prescribing of these medications or deprescribing of other high-risk medications. Patients with generalized anxiety or panic disorder or followed by specialists may also be included, but this would be non-differential. In addition, many of the tools are alert-based; while we hypothesize that incorporating behavioral science principles will reduce their likelihood of being ignored by providers, it is possible that the tools could increase alert fatigue [49]. However, the design will more explicitly identify tools that may unnecessarily increase alert fatigue and whether certain nudges work for certain types of providers.

## Discussion

Decision support in EHRs have shown promise in reducing high-risk prescribing for older adults, yet have not quite met that potential. While EHR strategies are widely used to support informational needs of providers, these tools have demonstrated only modest effectiveness at improving prescribing [49–53]. In a recent systematic review, 57% of studies found that decision support influenced provider behavior, yet effect sizes were small (i.e., mean change of < 5%), and many studies had a high-risk of bias [54]. EHR tools may currently be ineffective in part due to insufficient focus on factors behind clinical inertia, prescribing behavior, or workflow. To our knowledge, only three trials have specifically studied decision support for inappropriate prescribing in ambulatory older adults, which found modest effectiveness [20, 21, 52, 55]. Other decision supports, such as prescribing defaults, have not to our knowledge been applied to inappropriate prescribing in older adults [32, 56]. Discontinuing a medication may also prevent additional behavioral challenges, such as loss-aversion and endowment, compared with adding medication.

Once the trial is completed, these results will also need to be considered in relation to other efforts to reduce inappropriate prescribing in older adults. Limited prior evidence suggests that providing information alone via decision support at the time of a patient encounter could be insufficient at reducing prescribing on its own [20, 21, 55]. Other interventions that are not exclusively provider-facing, such as pharmacist interventions, including medication reviews, and in-person patient education, have also demonstrated some success in reducing inappropriate prescribing, yet these can be more resource intensive and may require actions outside of typical clinical workflow [11, 21, 57]. As the capabilities of EHRs increase, there still exists much opportunity to improve deprescribing efforts aimed at providers directly, including leveraging different time points in workflow and presenting different types of tools to facilitate deprescribing [58, 59].

Over the past decades, behavioral sciences have provided great insight into how to change behaviors by better understanding individuals’ underlying motivations, how they mentally “account” for various options, and processes necessary for sustained changed [44, 45]. These observations and application of their principles has effectively changed behavior in other settings and are likely applicable to EHR systems to improve the uptake of evidence-based care, such as improving prescribing in older adults [60]. The effectiveness of tools could be enhanced by leveraging principles of behavioral economics and related sciences, but they have had very limited application in EHRs and, more specifically, for prescribing in older adults.

Because there are numerous ways these tools could be designed and delivered using behavioral science, we are using an adaptive trial. Adaptive trials are increasingly emerging as options for increasing the efficiency and scale of interventions tested in clinical medicine than traditional parallel groups allow [61]. Accordingly, applying adaptive trials to delivering other healthcare interventions, such as in implementation research, would allow the possibility of testing faster and with more efficiency [36]. Because implementation research seeks to test how to promote uptake of evidence-based interventions and abandon strategies that are harmful, methods to generate evidence faster are central to the field. In specific, NUDGE-EHR will determine the components of EHR tools that are most impactful at changing provider behavior, which is fundamentally an implementation question. By using a study design that allows for the testing of different EHR implementation strategies, we will provide generalizable evidence both to healthcare practices about specific strategies that should be used in EHRs as well as to implementation researchers about the practicalities of how to test different delivery strategies simultaneously [62, 63].

Therefore, this overall approach, regardless of ultimate outcomes of the study itself, could be replicated by others to enhance the conduct and evaluation of pragmatic trials in implementation science. NUDGE-EHR will advance our understanding about how behavioral science can optimize clinical decision support to reduce inappropriate prescribing and improve patient health outcomes as well as how to use adaptive trial designs in healthcare delivery and implementation science.

## Supplementary Information

**Additional file 1: Supplement 1** Clinical trial protocol

**Additional file 2: Supplement 2, eFigure 1** Provider tapering information and talking points embedded in enhanced alerts. **Supplement 2, eFigure 2** SmartSet order set embedded within enhanced alerts. **Supplement 2, eFigure 3** Enhanced encounter opening alert used in Arms 2, 6, and 10. **Supplement 2, eFigure 4** Arm 3: Enhanced order entry alert + follow-up message. **Supplement 2, eFigure 5** Arm 7: Simplified enhanced order entry alert. **Supplement 2, eFigure 6** Arms 9 and 10: Sign-off approval alert that triggers to providers when electronically signing off on refill medications ordered by support staff. **Supplement 2, eFigure 7** Arm 11: Pre-commitment/consistency alert + enhanced order entry alert. **Supplement 2, eFigure 8** Arm 13: Enhanced order entry alert with different risk framing. **Supplement 2, eFigure 9** Arm 15: Non-enhanced order entry alert. **Supplement 2, eTable 1** Behavioral principles in electronic health record tools tested in regression model.

## Data Availability

Data sharing is not applicable to this article as no datasets were generated or analyzed during the current study.

## Abbreviations

BPA: Best practice advisory
EHR: Electronic health record
NUDGE-EHR: Novel uses of designs to guide provider engagement in electronic health records
PCP: Primary care provider
PRECIS-2: Pragmatic explanatory continuum indicator summary
Z-drug: Sedative hypnotic Z-drug
