# Supplementary material for: Rationale and design of the Novel Uses of adaptive Designs to Guide provider Engagement in Electronic Health Records (NUDGE-EHR) pragmatic adaptive randomized trial: a trial protocol
Source: Implement Sci. 2021 Jan 7;16:9. doi: 10.1186/s13012-020-01078-9 (PMC7792313; doi:10.1186/s13012-020-01078-9)
Supplement: Supplementary file 1 — Additional file 1: Supplement 1 Clinical trial protocol [file 13012_2020_1078_MOESM1_ESM.pdf]

CLINICAL TRIAL PROTOCOL

Optimizing electronic health record prompts with behavioral economics to improve prescribing for older adults: **Adaptive trial at Atrius Health**

Funded by the National Institute on Aging

Principal Investigators:
Niteesh Choudhry, MD, PhD
Julie Lauffenburger, PharmD, PhD

Brigham and Women's Hospital and Harvard Medical School
Center for Healthcare Delivery Sciences and
Division of Pharmacoepidemiology and Pharmacoeconomics

TABLE OF CONTENTS

SUMMARY OF CHANGES FROM PREVIOUS VERSION 4

1. BACKGROUND AND RATIONALE 5

2. STUDY AIMS 5

3. STUDY DESIGN 6

3.1 STUDY SITE 6
3.2 OVERALL DESIGN 7
3.2.1 Stage 1 Design 7
3.2.2 Stage 2 Design 8
3.3 STUDY SCHEMA 9
3.4 SCIENTIFIC RATIONALE FOR STUDY DESIGN 9
3.5 JUSTIFICATION FOR INTERVENTION 10
3.6 END-OF-STUDY DEFINITION 10
3.7 DATA SOURCES 11
3.8 SCHEDULE OF ACTIVITIES 11

4. STUDY POPULATION 12

4.1 INCLUSION CRITERIA 12
4.2 EXCLUSION CRITERIA 13
4.3 RECRUITMENT AND RETENTION 13
4.3.1 Informed consent considerations 13
4.3.2 Inclusivity of study subjects 14

5. STUDY INTERVENTIONS 14

5.1 THERAPEUTIC AREAS 14
5.2 STUDY INTERVENTIONS 14
5.3 MEASURES TO MINIMIZE BIAS: RANDOMIZATION AND BLINDING 17

6. STUDY ASSESSMENTS AND PROCEDURES 17

6.1 BASELINE DATA 17
6.2 OUTCOMES 18
6.3 ADVERSE EVENTS AND UNANTICIPATED PROBLEMS 19

7. STATISTICAL CONSIDERATIONS 22

7.1 STATISTICAL HYPOTHESES 22
7.2 SAMPLE SIZE DETERMINATION 22
7.3 STATISTICAL ANALYSES 22
7.3.1 ANALYSIS OF THE PRIMARY ENDPOINT 22
7.3.2 ANALYSIS OF SECONDARY ENDPOINTS 23
7.3.3 BASELINE DESCRIPTIVE ANALYSES 24
7.3.4 SUBGROUP ANALYSES 24
7.3.5 EXPLORATORY ANALYSES 24

8. ETHICAL AND REGULATORY REQUIREMENTS 25

8.1 ETHICAL CONDUCT 25
8.2 INFORMED CONSENT 27
8.3 CONFIDENTIALITY AND PRIVACY 27

8.4 SAFETY OVERSIGHT 29
8.5 BENEFIT RISK ASSESSMENT 30
8.5.1 KNOWN POTENTIAL RISKS 30
8.5.2 KNOWN POTENTIAL BENEFITS 30
8.5.3 ASSESSMENT OF POTENTIAL RISKS AND BENEFITS 31

9. LIST OF REFERENCES 31

Summary of Changes from Previous Version

Date of submission	Summary of Revisions Made	Rationale for modification	Approval date
12/19/2019	- Modified number of study arms from 15 to 16 and clarified intervention components<br>- Clarified randomization scheme and follow-up for Stage 1 and 2<br>- Provided final version of patient materials available as part of provider-facing interventions	- We are testing 14 different possible enhanced EHR alerts for providers in Stage 1, not 13 different possible alerts. We have also slightly modified the components included in the EHR tools for providers. We have included a new study schema accordingly.<br>- We are clarifying how we are planning to randomize providers in Stage 2<br>- We have finalized the customizable patient instructions included in the SmartSet order set for providers	1/7/2020
3/6/2020	- Updated to 206 providers based on newer baseline data<br>- Clarified randomization scheme for providers in Stage 2<br>- Provided newer study schema to reflect these changes<br>- Added in NCT number for the trial	- We clarified the number of providers based on inclusion/exclusion criteria, to more accurately reflect expected recruitment targets.<br>- We also refined the randomization plan for Stage 2.	3/27/2020
9/7/2020	- Updated Manual of Procedures<br>- Clarified expected sample size based on updated feasibility data	- These changes were made to reflect typographical modifications requested by the NIA and to ensure alignment across different documents in procedures for the study.	9/10/2020
9/29/2020	- Clarified that a telehealth visit is also considered an encounter<br>- Confirmed final expected sample size	- These changes were made to reflect discussions with statistician and DSMB members.	10/1/2020
11/2/2020	- Revised statistical nomenclature and stages of analyses	- These nomenclature changes were made to reflect refinements with statistician.	11/3/2020

1. Background and Rationale

The prescribing of inappropriate medications for older adults is extremely common in the United States, ranging from 12% in community settings to 40% of those who are institutionalized.[1-3] Benzodiazepines, anticholinergics, and sedative hypnotics are among the most commonly prescribed in circumstances that are inconsistent with practice guidelines.[3,4] While inappropriate prescribing increases the risk of adverse health consequences for all patients, older adults are particularly vulnerable.[2,5-7] Physicians' lack of awareness of alternatives, ambiguous practice guidelines, and perceived pressure of patients or caregivers are among the reasons why these drugs are used more than might be optimal.[2]

Reducing inappropriate use of these drugs may be achieved through decision support tools for physicians that are embedded in electronic health record (EHR) systems. While EHR strategies are widely used to support the informational needs of providers, these tools have demonstrated only modest effectiveness at improving prescribing.[8-13] The moderate effectiveness of current clinical decision support tools is thought to be largely due to what content they contain and the lack of provider-focused design principles being used to develop them.[14,15] Prior approaches have also been criticized for the sheer volume of alerts, the lack of clinical significance of the tools, and the poor/delayed timing of the clinical decision support (i.e., after the prescribing decision). Accordingly, the effectiveness of these tools could be enhanced by leveraging recently-gained insights from behavioral economics and other related sciences. Their application to EHRs has been limited, and they have not been used to reduce the prescribing of potentially harmful medications to older adults.

2. Study Aims

The overall goal of the proposed research is to evaluate whether EHR-based tools, optimized using behavioral science principles, reduce inappropriate prescribing among older adults. Our overall hypothesis is that thoughtful incorporation of behavioral principles into

EHRs will reduce inappropriate prescribing and adverse drug events among older adults compared to usual care.

The objectives and endpoints for this adaptive trial are summarized below.

OBJECTIVES	ENDPOINTS	JUSTIFICATION FOR ENDPOINTS
Primary		
To determine whether EHR-tools designed using behavioral science principles are more effective than at reducing inappropriate prescribing of high-risk medications in older adults than standard EHR tools or usual care.	Composite of 1) discontinuation of high-risk medications (benzodiazepines, sedative hypnotics, or anticholinergics [in secondary analyses]) or 2) ordering a gradual dose taper for one of these medications	These outcomes are rapidly measurable using EHR data alone and will provide evidence of provider behavior change.
Secondary		
To examine whether behavioral science-based EHR tools reduce cumulative prescribing of high-risk medications in older adults compared with usual care.	Quantity of high-risk medication prescribed, defined by number of milligram equivalents of high-risk medications prescribed to patients in follow-up	These outcomes capture the extent to which high-risk medications are cumulatively prescribed to patients by all Atrius providers over the follow-up period.
Tertiary/Exploratory		
To evaluate whether behavioral science-based EHR tools reduce the risk of clinically-significant adverse drug events, falls, fractures, hospitalizations, or emergency room visits compared with usual care.	Rates of adverse drug events, falls, fractures, hospitalizations, and emergency room visits in follow-up; quantity of high-risk medications dispensed to patients in follow-up period	These outcomes measure clinical outcomes that are consequences of these high-risk medications, measured in medical and pharmacy administrative claims data.

3. Study Design

3.1 Study site

This study will be conducted in outpatient and acute care practices of Atrius Health, a large integrated delivery network in eastern and central Massachusetts. Atrius has a fully functional EHR, EpicCare (www.epicsys.com), that supports computerized ordering of medications. Atrius is comprised of 29 clinical and multi-specialty outpatient locations with 875 physicians.

3.2 Overall design

We propose an open-label adaptive 2 Stage cluster-randomized, NIH-defined Stage III pragmatic trial to evaluate whether EHR-based tools designed using behavioral principles reduce inappropriate prescribing and adverse outcomes among older adults (See Study Schema in Section 3.3). We hypothesize that these tools will reduce prescribing of high-risk medications (primary outcome), cumulative prescribing of high-risk medications (secondary outcome), and clinically-significant adverse drug events like sedation and confusion (tertiary outcome) and cumulative utilization of high-risk medications (tertiary outcome) compared with usual care.

Because there are many ways in which the tools could be structured and delivered, we will use an adaptive trial design that involves two Stages of evaluation to rapidly identify which of many possible tools are most promising for changing provider behavior. Primary care providers will be the unit of randomization. All of the study sites will be at Atrius Health.

3.2.1 Stage 1 Design

In Stage 1 of the trial, we will randomize approximately 200 primary care providers at Atrius Health approximately equally to either usual care or active intervention. Of the half of providers randomized to active intervention, we will randomize them equally to one of 15 active intervention arms. Providers randomized to one of the 15 active intervention arms will receive an EHR tool to guide the care of their eligible patients. Providers randomized to usual care will receive no EHR tool, as is current clinical practice. Providers will be eligible for the trial if they prescribed a benzodiazepine or sedative hypnotic to at least one older adult in the 180 days prior to randomization. We will randomize providers within strata based on their clinic size and baseline rates of high-risk medication prescribing.

Eligible patients of these providers will be who meet the following criteria: 1) older adults (aged 65 years or more) and 2) who have been prescribed at least 90 pills of benzodiazepine or sedative hypnotic in the last 180 days.

After 6 months (dictated by the average number of observed data points, i.e., at least 1 eligible patient for whom the EHR tools fired per physician), an interim analysis of Stage 1 participants will be performed to rank the 15 active intervention arms based on their observed effect size at reducing prescribing of high-risk medications. Up to 5 of the most promising active intervention arms based on their effect size will be tested in Stage 2. If more than 5 are promising, we will choose the top 5. If 1 to 5 arms are promising, we will choose those for testing in Stage 2. If none are promising, we will combine active interventions within the arms based on the most effective factors. After this interim analysis, the Stage 1 providers in the "winning" arms (i.e., the promising arms) will be randomly assigned to continue to receive their original treatment assignments or to usual care to test holdover/persistency effects. Similarly, the Stage 1 providers assigned to treatment arms determined to be statistically inferior will be randomly assigned in equal proportions to one of the winning arms or to usual care.

3.2.2 Stage 2 Design

In Stage 2, we will randomize the primary care providers at Atrius Health who were assigned to usual care in Stage 1 in equal proportions to up to one of the 5 most promising treatment arms or to continue to receive usual care. We will also randomize any additional providers who prescribed at least one eligible anticholinergic to at least one older adult in the 180 days prior to randomization for secondary analyses. Providers randomized to one of the 5 selected treatment arms will receive an EHR tool to guide their care of eligible patients. As in Stage 1, we will randomize providers based on demographic characteristics, patient case-mix factors, and baseline rates of high-risk medication prescribing. Patient eligibility will be identical to that used in Stage 1 except patients will also be included if they had been prescribed at least 90 pills of eligible anticholinergics in the last 180 days. Follow-up in Stage 2 will last 8 months.

3.3 Study Schema

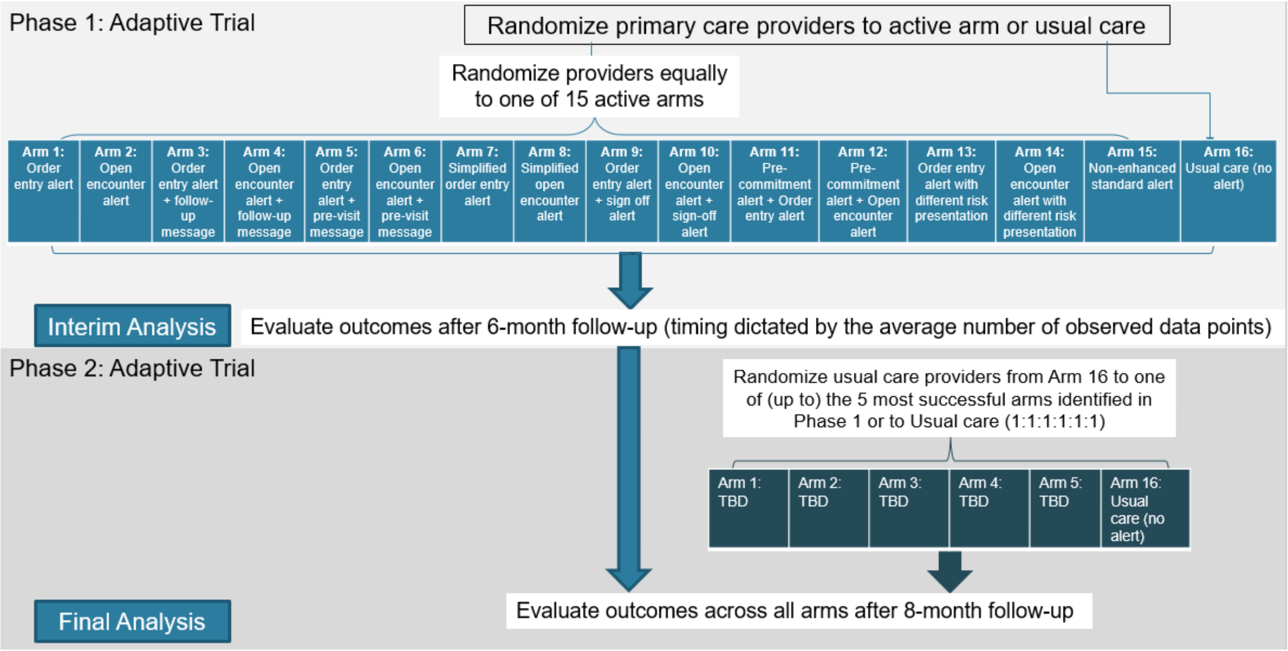

3.4 Scientific rationale for study design

Because there are numerous ways in which these EHR tools could be designed using behavioral principles, we propose a randomized adaptive design that will increase statistical efficacy.[16,17] This adaptive method has been used to improve the efficiency of traditional trials.[16-20] We propose to sequentially modify (i.e., adapt) the EHR tools that are presented to providers in the intervention groups based on identifying the tools that are the least and most successful at reducing inappropriate prescribing. This approach is highly feasible in the current context since the outcome on the basis of which intervention adaptation will occur (i.e., inappropriate prescribing) will be almost immediately observable using EHR data. Further, the use of a randomized trial in this setting is scientifically justified, as this design will be able to provide evidence of causality in the effectiveness of the tools on reducing prescribing and rates of clinical outcomes. An observational study design, by contrast, would not provide the same degree of scientific rigor.

3.5 Justification for intervention

The focus areas for the EHR tools will be primarily drawn from the outpatient Choosing Wisely recommendations in geriatric medicine but are also informed by the Beers Criteria and other major clinical guidelines.[21-23] In specific, we plan to focus on the following therapeutic classes: (1) benzodiazepines; (2) sedative hypnotics (sleep medicines) and (3) anticholinergics. These classes were chosen because they all have established clinical guidelines recommending reductions in use, continue to be heavily over-prescribed, contribute significantly to poor clinical outcomes in older adults, and also have non-drug or less risky therapeutic alternatives. While prescribing non-drug options may be the optimal alternative to these potentially inappropriate medications, their adverse effects could also be attenuated by choosing alternative medications in a different drug class, lower doses of medication, or alternative, safer medications within the same drug class.

We have chosen to focus on key principles of behavioral economics and cognitive psychology to “nudge” providers to optimize prescribing, including timing, salience, framing, simplification, cold state outreach, pre-commitment, and boostering. These principles were selected based on their effectiveness in other settings[24-27], their applicability to the care of older adults, and their ability to be adapted to the EHR context.

3.6 End-of-study definition

The trial will be completed 8 months after Stage 2 randomization. Providers and their eligible patients will be followed until the end of this follow-up date, or until censoring.

3.7 Data sources

We will use EHR data to implement the EHR tools, identify study subjects, track study progress, and evaluate the effect of the interventions. We will also use administrative claims data to evaluate tertiary outcomes among the subgroup of patients with claims data.

The Atrius data warehouses reside in an Oracle environment and consist of the Clarity and Payer databases. The Clarity database is a relational database that contains clinical and financial information from the Epic Suite of products; including the electronic medical record system, the appointment scheduling system, the patient accounting system, and the master patient index (Identity). The various tables within the Clarity database are refreshed on a daily, weekly or monthly basis. The Payer database contains medical claims, pharmacy claims and membership managed-care data from the payers with whom this organization has capitated contracts. The tables within the Payer database are refreshed monthly.

3.8 Schedule of activities

**Data collection**	**Stage 1: Pre-randomization**	**Stage 1: Follow-up**	**Stage 2: Pre-randomization**	**Stage 2: Follow-up**
EHR review for provider eligibility	X		X	
Patient characteristics (Demographics, clinical characteristics)	X		X	
Provider characteristics (Demographics, patient case-mix)	X		X	
Benzodiazepine/sedative hypnotic prescribing		X		X
Benzodiazepine/sedative hypnotic dispensations				X
Anticholinergic prescribing/dispensations				X
Adverse drug events		X		X
Falls or fractures				X
Resource use (hospitalizations, ER visits)				X

4. Study Population

The study will intervene upon primary care providers (primary care provider designated physicians, nurse practitioners, physician assistants) and their patients in the outpatient practices of Atrius Health.

4.1 Inclusion Criteria

The study will include provide and patient subjects. Limited inclusion criteria will be applied to maximize generalizability in accordance with pragmatic trial principles by PRECIS-2 (PRagmatic Explanatory Continuum Indicator Summary).[28] The study criteria are defined below.

<u>Stage 1</u>

Providers will be eligible for this Stage of the trial if they:

- are a primary care provider at Atrius Health
- prescribed a benzodiazepine or sedative hypnotic to at least one older adult (aged 65 years or more) in the prior 180 days

Patients will be included for this Stage of the trial if they:

- are the primary care patient of an eligible provider
- are aged 65 years or more
- have been prescribed at least 90 pills of benzodiazepine or sedative hypnotic in the prior 180 days

<u>Stage 2</u>

Providers will be eligible for this Stage of the trial if they:

- are a primary care provider at Atrius Health

- prescribed a benzodiazepine, sedative hypnotic, or anticholinergic to at least one older adult in the 180 days prior to randomization

Patients will be included for this Stage of the trial if they:

- are the primary care patient of an eligible provider
- are aged 65 years or more
- have been prescribed at least 90 pills of benzodiazepine or sedative hypnotic or at least 90 pills of an eligible anticholinergic in the prior 180 days

4.2 Exclusion Criteria

Patients not meeting the inclusion criteria above will not be included in the study. No other exclusion criteria will be used.

4.3 Recruitment and retention

4.3.1 Informed consent considerations

We have received a waiver of informed consent and HIPAA authorization for all physician-subjects and patient-subjects in this study. The goal of this project is to improve existing decision support to reduce the use of potentially dangerous medications in the elderly, consistent with numerous professional guidelines and quality metrics. Providers will retain oversight of their patients' care and will be able to make therapeutic choices based using their professional judgement. Patients will not receive any direct intervention as a result of their inclusion in the study. We have provided further information in Section 8.2.

4.3.2 Inclusivity of study subjects

Physician and patient subjects will be included based on their meeting eligibility criteria as part of routine care, and the study population will be highly inclusive. Further, pilot data indicate that 201 primary care providers at Atrius currently meet eligibility criteria. Based on these same pilot data, the number of patients included in the analyzed population should exceed 2000 patients in Stage 1 and 4000 patients in Stage 2. Importantly, we expect these participants to cover a broad range of participants by gender and race/ethnicity. Of these, 59.2% are female, 84.6% are White, 6.4% are Black or African American, 1.4% are Hispanic, 3.1% are Asian/Pacific Islander, 0.3% are American Indian/Native American, and 4.2% are other races or unknown. These estimates at Atrius Health are similar to the overall estimates of older adults in the U.S. Medicare population. Further, given the minimal risk nature of the study, participants will not receive incentives, remuneration, or be required to provide informed consent.

5. Study Interventions

5.1 Therapeutic areas

The focus areas for the EHR tools will be primarily drawn from the outpatient Choosing Wisely recommendations in geriatric medicine but are also informed by the Beers Criteria and other major clinical guidelines.[4,29] In specific, we plan to focus on the following therapeutic classes: (1) benzodiazepines; (2) sedative hypnotics (sleep medicines) and (3) anticholinergics.

5.2 Study interventions

Physicians randomized to one of the 15 active intervention arms will receive one of several possible enhanced EHR decision support tools to guide their care of eligible patients

when these patients come to the clinic for an appointment. The type and timing of an alert will vary based on the intervention arm.

These EHR tools will be tested in combination or on their own in the 15 active intervention arms outlined below:

	Components included in the EHR tools							
**Arm**	Alert type	Order entry (X) vs. Open encounter	Follow-up booster	Cold state outreach	Simplification	Sign-off moment	Pre-commitment	Framing: Guideline risks
**1**	Enhanced	X						
**2**	Enhanced							
**3**	Enhanced	X	X					
**4**	Enhanced		X					
**5**	Enhanced	X		X				
**6**	Enhanced			X				
**7**	Enhanced	X			X			
**8**	Enhanced				X			
**9**	Enhanced	X				X		
**10**	Enhanced					X		
**11**	Enhanced	X					X	
**12**	Enhanced						X	
**13**	Enhanced	X						X
**14**	Enhanced							X
**15**	Basic	X						
**16**	None							

Arms 1 through 14 are enhanced EHR tools to encourage the deprescribing of the medications under study. Arm 15 is a basic EHR alert that is meant to represent the type of clinical decision support that is commonly given to providers – without enhancements using behavioral science principles (if any decision support is provided). Physicians randomized to usual care (Arm 16) will receive no intervention.

The central component of arms 1 through 14 will be an enhanced EHR alert (known as a Best Practice Advisory [BPA]). The enhanced BPA will appear on each provider's EHR screen and will contain several standard components. The BPA will:

1. give providers information about why the medication is dangerous for their patient using the behavioral science principle of salience to make this information as impactful as possible;

2. include a set of tips to help providers discuss medication discontinuation with their patients;
3. ask providers to select an acknowledgment reason if they decided not to discontinue the medication;
4. include a SmartSet order set that will allow providers to order a gradual dose taper for their patient, which limits risks of withdrawal symptoms for the patients for benzodiazepines and sedative hypnotics, order alternative medications, place a referral to a behavioral health specialist, provide instructions on how to make lifestyle modifications to improve patient symptoms, and add customizable patient instructions for how to gradually taper off benzodiazepines and sedative hypnotics, as applicable.

The BPA will display either when the provider orders a medication (Order Entry) or opens the chart (Open Encounter) for eligible patients, depending on the arm. In specific, the enhanced BPA will fire at Order Entry in Arms 1, 3, 5, 7, 9, 11, 13 and 15; the enhanced BPA will fire at Open Encounter in Arms 2, 4, 6, 8, 10, 12 and 14.

We will also test several other modifications to this enhanced BPA. In specific, as outlined in the table above, we will add in a boostering option in the enhanced BPA in Arms 3 and 4, which is a provider-directed option for a follow-up in-basket message sent 4 weeks after the BPA is triggered. In Arms 5 and 6, we will incorporate a prior, “cold state” outreach component, consisting of an in-basket message sent to the eligible provider 2 days before the eligible patient is scheduled for an in-person or telehealth visit. Arms 7 and 8 will test simplified language of the BPA. Arms 9 and 10 will test the addition of a BPA at medication sign-off for providers. Arms 11 and 12 will test the use of a two-staged pre-commitment BPA in which the providers are prompted to discuss risks of these high-risk medications and share a handout about the risks with their patients, at their own discretion. Arms 13 and 14 will test the framing of different risks of the high-risk medications in the BPA.

If patients are eligible for alerts to be fired for multiple therapeutic classes of interest (e.g., benzodiazepines and sedative hypnotics), the EHR tools will appear for both classes separately.

5.3 Measures to minimize bias: randomization and blinding

Providers will be randomized to treatment arms in equal proportions. We will use provider-based cluster randomization to minimize the possibility of contamination in study interventions between practices and clinic staff. For the randomization, we will use stratified randomization based on clinic practice size and baseline rates of prescribing to reduce potential imbalances between the providers assigned to the treatment arms.

The providers will not be blinded to which arm they were assigned to, as blinding is the context of an intervention that is intended to motivate action will be infeasible. The study statistician, in partnership with data analysts at Atrius Health, will generate and implement the randomization scheme, with oversight by the Principal Investigators. Investigators will be blinded to the treatment arms during interim and final analyses.

6. Study Assessments and Procedures

6.1 Baseline data

We will collect baseline data on patients and providers using extracted EHR data and/or administrative claims data as applicable for the study aim. This baseline data will be used to assess any potential imbalances in the characteristics of providers or patients despite randomization. The baseline data will include, but are not limited to: gender, rural/urban practice setting, baseline rates of prescribing, practice location, and patient case-mix. We will also collect patient data that include but are not limited to: sociodemographic data, medical

history and comorbidities, baseline resource utilization in prior 12 months (i.e., number of visits), biometric values (e.g., serum creatinine, systolic/diastolic blood pressures).

6.2 Outcomes

For both Stages, the primary outcome will be a binary composite measure of a reduction in inappropriate prescribing, evaluated using EHR data from Atrius Health. In specific, we will measure a composite of 1) discontinuation of high-risk medications (benzodiazepines, sedative hypnotics, or anticholinergics [included in secondary analyses]) or 2) ordering a gradual dose taper (for benzodiazepine or sedative hypnotics). If either of these actions is taken by the provider for a specific patient at any point in the follow-up window, we will classify the patient as having had a reduction in inappropriate prescribing. If the patient has multiple therapeutic classes of interest (e.g., benzodiazepines and sedative hypnotics), we will classify patients with a reduction for any class as a "reduction" for the composite measure. In secondary analyses, we will include anticholinergics. We will also stratify patients by their number of eligible therapeutic classes (i.e., one, two, or three classes) and analyze outcomes within these strata.

In Stage 2, secondary outcomes include the quantity of high-risk medication prescribed, defined by number of milligram equivalents of high-risk medications prescribed to patients in the follow-up period to capture cumulative prescribing by all providers at Atrius Health. As above, we will also stratify patients by their number of eligible therapeutic classes (i.e., one, two, or three classes) and analyze outcomes within these strata.

In Stage 2, tertiary outcomes will include the extent to which medications are filled and consumed by patients, as measured within the subgroup of patients with claims data. In specific, these outcomes will be conducted among the large subgroup of patients for whom Atrius receives administrative claims data, including pharmacy claims, from their insurer. In particular, we will measure the quantity of high-risk medication dispensed, defined by number

of milligram equivalents of high-risk medications filled by patients, in follow-up, using pharmacy claims data. Other tertiary outcomes will include the occurrence of clinically-significant adverse drug events, including but not limited to, sedation or cognitive impairment, and all-cause hospitalizations and falls or fractures, measured in administrative claims data. These clinical outcomes will be evaluated using validated and CMS-driven ICD-10-CM diagnosis and procedure-based algorithms applied to these patients' medical and pharmacy administrative claims data. Because the sensitivity of clinical outcomes in EHR systems is known to be low (e.g., because patients may seek subsequent care at other healthcare systems), using routinely-collected data from insurers overcomes this limitation.[30]

6.3 Adverse events and unanticipated problems

For provider-subjects, the EHR decision support designed for this trial is only meant to highlight information that could be useful in patient management and prescribing. Therefore, we do not anticipate any safety issues to arise with regards to provider-subjects who receive the electronic decision support, and the IRBs who have reviewed our prior proposals have agreed with this general approach.

For patient-subjects, we do not anticipate the occurrence of any adverse events as a result of providers receiving decision support aimed at reducing the use of potentially unsafe medications that already have established clinical guidelines advising against their use. The decision support provides resources to help patients safely discontinue the high-risk medications under study (e.g., providing tapering guidelines and facilitating the substitution of lower-risk therapies). There is a theoretical risk of precipitating withdrawal, but the risk is less than the continued risks of ongoing use of the drugs being addressed by the interventions (See Section 8.5 for further detail).

We will ensure the safety of patient-subjects by leaving ultimate clinical decision-making in the hands of the evaluating provider who is in charge of caring for the patient. The study

team will not be providing any direct care to patients, and all treatment decisions will ultimately be made by the patients' own medical teams. As a result, any adverse events will be handled in the course of regular clinical care. Further, to maximize the generalizability of the results and to avoid co-intervention, patients will not be required to have study-specific monitoring as part of the proposed pragmatic trials. Therefore, we do not plan to use any patient-directed prospective monitoring of Adverse Events (AEs) or Significant Adverse Events (SAEs) in this trial. An *Adverse Event (AE)* is defined as any untoward or unfavorable medical occurrence in a human study participant, including any abnormal sign (e.g. abnormal physical exam or laboratory finding), symptom, or disease, temporally associated with the participants' involvement in the research, whether or not considered related to participation in the research.

- Adverse Events will be classified using the following rating scales:
  - Severity: Mild, Moderate or Severe
    - Mild: Awareness of signs or symptoms but are easily tolerated
    - Moderate: Events introduce a low level of inconvenience or concern but may interfere with daily activities but are usually improved by simple therapeutic measures
    - Severe: Events interrupt the participants' normal daily activities and generally require systemic drug therapy
  - Expectedness: Unexpected or Expected
    - Unexpected: nature or severity of the event is not consistent with the condition under study
    - Expected: event is known to be associated with the intervention or condition under study.

Serious Adverse Event (SAE) are defined as any adverse event that results in death, is life threatening, or places the participant at immediate risk of death from the event as it occurred, requires or prolongs hospitalization, causes persistent or significant disability or

incapacity, results in congenital anomalies or birth defects, and is another condition which investigators judge to represent significant hazards.

However, our plan for data and safety monitoring does include multiple mechanisms to ensure minimal risk of participation in the trials. We will leverage an automatic adverse event reporting and review system to observe and monitor for any SAEs that do occur. In specific, providers report adverse events through an online reporting system. All reports are routinely reviewed by quality and safety specialists at Atrius. Atrius, under the oversight of our Site PI, will use these reports to monitor for AEs and SAEs throughout the course of the study. Any reports of deaths will be submitted to the NIA Program Officer and to the Data Safety Monitoring Board (DSMB) Chair or designated DSMB member within 24 hours. Any unanticipated SAEs deemed by the specialists and Site PI at Atrius to be related to the intervention will be reported to the NIA PO and to the DSMB Chair or to the designated DSMB member within 48 hours of the study's knowledge of the SAE. All other reported SAEs and AEs received by the study team will be reported to the NIA Program Officer and to the DSMB quarterly, unless otherwise requested by the DSMB or a Safety Officer.

7. Statistical Considerations

7.1 Statistical Hypotheses

Our null hypothesis will be that rates of provider prescribing (defined by evidence of a reduction in prescribing of high-risk medication) in any one of the intervention groups will be no different than in the other arms, including the usual care arm.

7.2 Sample size determination

We powered the study for 15 active intervention arms with the following assumptions. We assumed a baseline rate of the composite outcome of 5% (i.e., that 5% of patients would have a medication discontinued or a taper ordered in the follow-up window), an intervention effect size of 15% (i.e., Odds ratio of 1.15 of discontinuation compared with usual care), alpha=0.05, power=0.8, and patient correlation of 0.3 within randomized providers. We also assumed an average cluster size of 20 patients per provider based on pilot data.

7.3 Statistical analyses

7.3.1 Analysis of the primary endpoint

The unit of analysis is at the patient-level. Therefore, for the primary outcome, we will use a generalized linear mixed model for binary outcomes to adjust for physician-level clustering and multiple patient observations per physician. The study arms will be analyzed using a multivariable regression model, in which each of features of the EHR tools will be included as covariates in an overall regression model that compares the effect of receiving an enhanced EHR decision support tool compared with usual care. In specific, these features will include: enhanced BPA (yes=1, no=0), timing of EHR tool (open encounter=1, order entry=0), whether a follow-up alert is enabled as part of the tool (yes=1, no=0), cold state outreach is used (yes=1, no=0), simplification is used (yes=1, no=0), sign-off is used (yes=1, no=0), pre-commitment is used (yes=1, no=0) or if different risks of the high-risk medications are

presented within the EHR tool (yes=1, no=0). In this way, we will observe and report an overall effect of the enhanced EHR tool compared with usual care as well as the effect of individual features through their coefficients in the model. We will conduct this interim analysis once at the end of follow-up in Stage 1 to determine the arms with the most potential promise. In specific, the 15 active arms will be ranked based on their observed effect size at reducing prescribing of high-risk medications from the covariate coefficients from the models. At the end of follow-up in Stage 1, we will also use these regression models to explore whether any of the intervention arms are inferior to usual care. We will repeat these analyses at the end of Stage 2 using all available data to determine whether any of the intervention arms were more effective than usual care at reducing prescribing of high-risk medications. In secondary analyses, we will include anticholinergics. The results from Stage 2 will be used as the definitive results for effectiveness of the EHR tools.

Because this is a randomized trial, our primary analyses are planned as unadjusted; however, if there are strong patient-level predictors of the outcomes not balanced by stratified randomization, we will adjust for these in the primary analyses. Given the nature of the data and how the outcomes are categorized, there will not be missing values for the primary endpoint, as the absence of action is classified as no action by the provider. For the primary analysis, we will include all eligible patients in the denominator who had at least one visit with their primary care provider. In secondary analyses, we will include all eligible patients of those primary care providers in the denominator, regardless of whether the patient visited the provider over the follow-up period.

7.3.2 Analysis of secondary endpoints

For the secondary outcome of cumulative medication prescribing, will use an identity link function and normally distributed errors within the generalized linear models. For tertiary adverse clinical outcomes and resource utilization outcomes, we will use a log link function and

binary distributed errors within the overall multivariable models. These models generate the estimated relative risks (RRs) with robust standard errors and are considered to be particularly appropriate when outcomes are common (e.g., incidences of ≥10%). For these outcomes from claims data, we will follow patients whose providers are randomized to the study from the time of randomization until they are censored due to lose of continuous enrollment in their health plan or leaving Atrius Health. Due to the nature of the randomization, we do not anticipate any systematic differences in the amount of follow-up time per arm but will account for any imbalances using inverse probability censoring weights.

Because this is a randomized trial, our primary analyses are planned as unadjusted; however, if there are strong patient-level predictors of the outcomes not balanced by stratified randomization, we will adjust for these in the primary analyses. Given the nature of the data and how the outcomes are being measured, there should not be missing values. However, should there be sufficient missing data (e.g., >10%), we will use multiple imputation.[31]

7.3.3 Baseline descriptive analyses

We will report the means and frequencies of pre-randomization variables separately for intervention and control subjects. Comparisons of these values will be performed using t-tests and chi square tests and their non-parametric analogs, as appropriate. The outcomes will be evaluated using intention-to-treat principles among all randomized patients.

7.3.4 Subgroup analyses

In subgroup analyses, we will explore whether there were any modifiers of the effects of the EHR tools. For example, we will explore if certain types of providers (e.g., by specialty) were more likely to respond to the EHR tools or if there were observable differences in patients who were less likely to receive inappropriate medications, such as gender or race/ethnicity.

7.3.5 Exploratory analyses

In secondary analyses, we will control for potential confounders which will be measured using EHR data from structured fields and administrative claims data from the Atrius data warehouse. These variables will include provider characteristics (such as specialty, age, and gender), patient characteristics (such as major comorbidities, race/ethnicity, and age), and practice characteristics (such as practice size).

8. Ethical and regulatory requirements

8.1 Ethical conduct

General oversight of the project by the principal investigators (Drs. Choudhry and Lauffenburger) will occur throughout the study period, including regular contact with practice managers and clinical leadership at each health center to obtain ongoing feedback. In addition, this protocol will undergo Institutional Review Board (IRB) evaluation by a centralized IRB for this multi-site clinical trial. Study data will be accessible at all times for the principal investigators (Drs. Choudhry and Lauffenburger) and co-investigators to review, if applicable. The principal investigators will review study conduct (e.g., protocol deviations) on a monthly basis. The principal investigators will also ensure that all protocol deviations for the trials are reported to the NIH and the IRB according to the applicable regulatory requirements.

We believe that the risks to participation for both sets of subjects (i.e. providers and patients) are no more than minimal for several reasons.

First, the intervention aims to emphasize guideline-recommended information for providers to assist in their decision-making when caring for older patients. Second, all treatment decisions will ultimately be made by licensed health care providers. Finally, the intervention is specifically provider-focused and delivered in an electronic health record system using information already available to providers. We believe there is no more than minimal risk involved to the provider subjects, as the providers will simply be “nudged” to alter their behaviors towards guideline recommended care, as opposed to being forced to do so. All

medical decisions are ultimately made by the provider. This trial will not interfere with the ordinary workings of the outpatient centers.

There is a small risk associated with altering medication prescribing behaviors, including allergic reactions or other adverse medication effects; however, these risks are no more than are encountered during routine clinical care and are less than patients would otherwise encounter if there were to receive the high-risk medications whose use the intervention seeks to reduce. In addition, these risks will be minimized in our protocol as we are relying on the provider to prescribe as they see best for their patient; the prescribing changes in the EHR tools are simply suggestions, not rigid rules for the providers. In addition, as described above, in the unexpected situation in which the EHR tools lead to worse prescribing decisions, we will discontinue those arms at the end of Stage 1.

The primary risk to patients will be privacy of health information. We will minimize the risk to privacy by taking appropriate steps to limit access to data to study investigators. Clinical data on the care for patients will be retrieved from the electronic medical records and insurer administrative claims at Atrius Health. The data extracts obtained from the electronic medical record and these claims are continuously used by Atrius clinical operations staff for quality assessment and improvement, and undergo routine, rigorous peer-review by experienced data analysts to ensure accuracy and completeness. Drs. Choudhry and Lauffenburger will work with the research project staff to ensure the accuracy of these data throughout the study period. For the purpose of conducting analyses of the study outcomes, this will involve creating scrambled patient and provider identifiers and sharing only limited Protected Health Information (PHI) with investigators for the purposes of analysis. The link between the identifiers and the medical record number will remain at Atrius Health in a password protected file. All team members have received appropriate training in data privacy.

8.2 Informed consent

We will enroll provider-subjects based on their being employed by Atrius Health as an outpatient primary care provider. As with other minimal-risk, quality improvement studies we have performed that involve electronic alerts to providers, formal informed consent will not be sought. First, the nature of this quality improvement intervention involves testing EHR decision support directly for providers (using information already available to them and a similar infrastructure they use in the course of regular clinical care). Second, the ability to understand the true effect of the intervention as it is delivered in the real world would be difficult to ascertain if true informed consent was sought. Third, obtaining true informed consent would predictably reduce the number of patients participating in the study, especially those from unrepresented populations, and therefore undermine the generalizability of the study results, a foundational aspect of pragmatic clinical trial principles. Fourth, this approach has been approved by clinical leadership at the health organization. In our prior work at these institutions, we have received a waiver of informed consent from the IRBs of these organizations for similar interventions. We also request a HIPAA waiver of patient authorization to access the administrative claims and EHR data necessary for outcome evaluation, as doing so would be impractical and infeasible to conduct the study.

While providers and patients will not be consented into the study, an organization-wide announcement will be circulated across Atrius to inform providers of the launch of an intervention leveraging clinical decision support tools to support improved prescribing for older adults.

8.3 Confidentiality and privacy

To protect against the risk of inappropriate disclosure of personal health information, the investigators at BWH will only receive Atrius with encrypted identifiers. The study team will also use limited PHI data for the purpose of analyzing the study. These analyses will be overseen

and conducted by Partners investigators. Atrius will disclose HIPAA-limited datasets encrypted by a study key only known to Atrius to the investigators to conduct the analyses. These datasets will consist of pharmacy and medical claims, laboratory information, and structured information from the EHR. The only PHI that will be shared with the study investigators are dates (e.g., date of birth, admission/discharge dates, and dates of medication fills) and zip code. Sharing this information will be necessary to assess the impact of the interventions.

We have a history of collaborative evaluations between these organizations and BWH that involves transfer of the minimum data necessary to complete rigorous evaluations, involving the use of encrypted identifiers to ensure patient confidentiality. The electronic data stored at Atrius will be safeguarded by state-of-the-art security protocols. The facilities have 24-hour security and are protected by locked entrances. Both health systems have computer networks in place that employ up to date virus protection software and enable password-protected access only to study investigators. All data transfers between the organizations will be accomplished using secure file transfer protocols. To ensure the confidentiality and security of all data, the research team operates a secure, state-of-the-art computing facility housed at Partners Healthcare's data center. The Partners data center is a secure facility that houses both computing environments as well as clinical systems and electronic medical records for several large hospitals in Eastern Massachusetts. Entry into the computer room requires staffed computer room security. The Division's computers are connected to the Partners networking backbone with 10 gigabit-per-second fiber links. Network security is overseen by electronic medical records systems to the research team's data. All data are transmitted to programmers' workstations in an encrypted state. Backups are created using the current Department of Defense standard for data security and are stored in a locked facility. The redundancy, extensive data power, and security of our computer facility confirm our capacity to collect and manage data and ensure confidentiality for all project participants.

As described, all members of the research team have completed or will complete appropriate human subjects research training and patient privacy training related to the Health Insurance Portability and Accountability Act (HIPAA). The setup for analysis of these HIPAA-limited data will be exactly the same as all of the other IRB applications that our Partners research division submits for secondary use of data. In fact, we have an umbrella-approval place in place with the Partners IRB for using these types of HIPAA-limited data. All of the datasets, including limited PHI, will be stored only on secure servers at Partners Healthcare's data center and will only be accessed by a limited number of individuals in the study team from this division who are all trained in data security and patient privacy.

8.4 Safety oversight

We plan to use a centralized Institutional Review Board (IRB) and a Data Safety Monitoring Board (DSMB) for all aspects of this research. We will also establish an independent data and safety monitoring board (DSMB) with experience in quality of care, patient safety, and biostatistics. The DSMB will act in an advisory capacity to monitor participant safety and evaluate the progress of the study, review procedures and management of the study. As previously described above, Atrius Health does not have its own IRB and cedes review to Partners' IRB. The DSMB reports will be shared with the local site PI within 72 hours of their completion. Drs. Choudhry and Lauffenburger are the PIs at Partners. The DSMB will consist of individuals with experience in quality of care, patient safety, and biostatistics. This committee will convene biannually and review data related to the study protocols and ensure protection of patient confidentiality and safety, as well as to monitor the quality of the data collected via the study protocols on a semi-annual basis. We will also be in routine contact with clinical leadership to obtain any feedback from clinicians regarding the studies. Compliance of regulatory documents and study data accuracy and completeness will be maintained through an internal study team quality assurance process. At each meeting, the

DSMB will make recommendations as to whether the studies should continue or if changes to the protocol are necessary for continuation. This trial will be registered with clinicaltrials.gov.

8.5 Benefit risk assessment

8.5.1 Known potential risks

There is a small risk associated with altering medication prescribing behaviors, including allergic reactions or other adverse medication effects; however, these risks are no more than are encountered during routine clinical care. In addition, these risks will be minimized in our protocol as we are relying on the provider to prescribe as they see best for their patient; the prescribing changes in the EHR tools are simply suggestions, not rigid rules for the providers. In the intervention arms, providers will be encouraged to follow national guidelines in the care of their patients and discontinue dangerous medications. It is recommended that some of these medications (benzodiazepines, sedative hypnotics) be discontinued through a gradual dose taper so as to avoid withdrawal symptoms, and suggested tapers will be provided as a solution within the EHR prompts. Another potential small risk to patients will be privacy of health information. We will minimize the risk to privacy by taking appropriate steps to limit access to data to study investigators. Clinical data on the care for patients will be retrieved from the electronic medical records and insurer administrative claims at Atrius Health.

8.5.2 Known potential benefits

This study is designed to improve electronic health record prescribing tools for providers caring for older adults. Potential benefits for participants in this study include improved decision support tools and guideline-concordant prescribing. The human subjects may benefit from discontinuing a dangerous drug that is not recommended for them. Additionally, the subjects and society may benefit in the future from accumulated knowledge that originates from this

research. We will also produce several EHR tool deliverables for this work for the public, researchers, and policymakers, which will be shared as generalized knowledge.

8.5.3 Assessment of potential risks and benefits

The intervention aims to emphasize guideline-recommended information for providers to assist in their decision-making when caring for older patients. All treatment decisions will ultimately be made by licensed health care providers. The intervention is specifically provider-focused and delivered in an electronic health record system using information already available to providers. We believe there is no more than minimal risk involved to the provider subjects, as the providers will simply be “nudged” to alter their behaviors towards guideline recommended care, as opposed to being forced to do so. All medical decisions are ultimately made by the provider. This trial will not interfere with the ordinary workings of the outpatient centers.

The potential societal benefits outweigh the minimal risk, especially in light of multiple measures in place to protect confidentiality. The data extracts obtained from the electronic medical record and these claims are continuously used by Atrius clinical operations staff for quality assessment and improvement, and undergo routine, rigorous peer-review by experienced data analysts to ensure accuracy and completeness. For the purpose of conducting analyses of the study outcomes, this will involve creating scrambled patient and provider identifiers and sharing only limited Protected Health Information (PHI) with investigators for the purposes of analysis. Because our intervention encourages providers to discontinue dangerous medications in a way that prioritized patient safety and enables the provider to retain full decision-making power of the care of the patient, there is no more than minimal risk involved for our patient and physician-subjects.

9. List of references

1. Zhan C, Sangl J, Bierman AS, et al. Potentially inappropriate medication use in the community-dwelling elderly: findings from the 1996 Medical Expenditure Panel Survey. *Jama.* 2001;286(22):2823-2829.
2. Zhang YJ, Liu WW, Wang JB, Guo JJ. Potentially inappropriate medication use among older adults in the USA in 2007. *Age and ageing.* 2011;40(3):398-401.
3. Guaraldo L, Cano FG, Damasceno GS, Rozenfeld S. Inappropriate medication use among the elderly: a systematic review of administrative databases. *BMC geriatrics.* 2011;11:79.
4. By the American Geriatrics Society Beers Criteria Update Expert P. American Geriatrics Society 2015 Updated Beers Criteria for Potentially Inappropriate Medication Use in Older Adults. *Journal of the American Geriatrics Society.* 2015;63(11):2227-2246.
5. Kim DH, Brown RT, Ding EL, Kiel DP, Berry SD. Dementia medications and risk of falls, syncope, and related adverse events: meta-analysis of randomized controlled trials. *Journal of the American Geriatrics Society.* 2011;59(6):1019-1031.
6. Peterson JF, Kripalani S, Danciu I, et al. Electronic surveillance and pharmacist intervention for vulnerable older inpatients on high-risk medication regimens. *Journal of the American Geriatrics Society.* 2014;62(11):2148-2152.
7. Kim DH, Newman AB, Lipsitz LA. Prediction of severe, persistent activity-of-daily-living disability in older adults. *American journal of epidemiology.* 2013;178(7):1085-1093.
8. Sequist TD, Morong SM, Marston A, et al. Electronic risk alerts to improve primary care management of chest pain: a randomized, controlled trial. *Journal of general internal medicine.* 2012;27(4):438-444.
9. Field TS, Rochon P, Lee M, Gavendo L, Baril JL, Gurwitz JH. Computerized clinical decision support during medication ordering for long-term care residents with renal insufficiency. *Journal of the American Medical Informatics Association : JAMIA.* 2009;16(4):480-485.
10. Steele AW, Eisert S, Witter J, et al. The effect of automated alerts on provider ordering behavior in an outpatient setting. *PLoS medicine.* 2005;2(9):e255.
11. Tamblyn R, Huang A, Perreault R, et al. The medical office of the 21st century (MOXXI): effectiveness of computerized decision-making support in reducing inappropriate prescribing in primary care. *CMAJ : Canadian Medical Association journal = journal de l'Association medicale canadienne.* 2003;169(6):549-556.
12. Embi PJ, Leonard AC. Evaluating alert fatigue over time to EHR-based clinical trial alerts: findings from a randomized controlled study. *Journal of the American Medical Informatics Association : JAMIA.* 2012;19(e1):e145-148.
13. Pell JM, Cheung D, Jones MA, Cumbler E. Don't fuel the fire: decreasing intravenous haloperidol use in high risk patients via a customized electronic alert. *Journal of the American Medical Informatics Association : JAMIA.* 2014;21(6):1109-1112.
14. Litvin CB, Davis KS, Moran WP, Iverson PJ, Zhao Y, Zapka J. The use of clinical decision-support tools to facilitate geriatric education. *Journal of the American Geriatrics Society.* 2012;60(6):1145-1149.
15. McCoy AB, Waitman LR, Lewis JB, et al. A framework for evaluating the appropriateness of clinical decision support alerts and responses. *Journal of the American Medical Informatics Association : JAMIA.* 2012;19(3):346-352.
16. Bhatt DL, Mehta C. Adaptive Designs for Clinical Trials. *The New England journal of medicine.* 2016;375(1):65-74.
17. Brown CH, Ten Have TR, Jo B, et al. Adaptive designs for randomized trials in public health. *Annu Rev Public Health.* 2009;30:1-25.
18. Cellamare M, Milstein M, Ventz S, Baudin E, Trippa L, Mitnick CD. Bayesian adaptive randomization in a clinical trial to identify new regimens for MDR-TB: the endTB trial. *Int J Tuberc Lung Dis.* 2016;20(12):8-12.

19. Goel S, Pernas S, Tan-Wasielewski Z, et al. Ribociclib Plus Trastuzumab in Advanced HER2-Positive Breast Cancer: Results of a Stage 1b/2 Trial. *Clin Breast Cancer.* 2019.
20. Trippa L, Lee EQ, Wen PY, et al. Bayesian adaptive randomized trial design for patients with recurrent glioblastoma. *J Clin Oncol.* 2012;30(26):3258-3263.
21. Panel AGSBCUE. American Geriatrics Society 2015 Updated Beers Criteria for Potentially Inappropriate Medication Use in Older Adults. *Journal of the American Geriatrics Society.* 2015;63(11):2227-2246.
22. O'Mahony D, O'Sullivan D, Byrne S, O'Connor MN, Ryan C, Gallagher P. STOPP/START criteria for potentially inappropriate prescribing in older people: version 2. *Age Ageing.* 2015;44(2):213-218.
23. Kuhn-Thiel AM, Weiss C, Wehling M, members Faep. Consensus validation of the FORTA (Fit fOR The Aged) List: a clinical tool for increasing the appropriateness of pharmacotherapy in the elderly. *Drugs Aging.* 2014;31(2):131-140.
24. Keller PA. Affect, Framing, and Persuasian. *Journal of Marketing Research.* 2003;40(1):54-64.
25. Yokum D, Lauffenburger JC, Ghazinouri R, Choudhry NK. Letters designed with behavioural science increase influenza vaccination in Medicare beneficiaries. *Nature Human Behaviour.* 2018;2(10):743-749.
26. Emanuel EJ, Ubel PA, Kessler JB, et al. Using Behavioral Economics to Design Physician Incentives That Deliver High-Value Care. *Annals of internal medicine.* 2016;164(2):114-119.
27. Purnell JQ, Thompson T, Kreuter MW, McBride TD. Behavioral economics: "nudging" underserved populations to be screened for cancer. *Preventing chronic disease.* 2015;12:E06.
28. Thorpe KE, Zwarenstein M, Oxman AD, et al. A pragmatic-explanatory continuum indicator summary (PRECIS): a tool to help trial designers. *CMAJ : Canadian Medical Association journal = journal de l'Association medicale canadienne.* 2009;180(10):E47-57.
29. McCormick WC. Revised AGS Choosing Wisely((R)) list: changes to help guide older adult care conversations. *Journal of gerontological nursing.* 2015;41(5):49-50.
30. Weiskopf NG, Weng C. Methods and dimensions of electronic health record data quality assessment: enabling reuse for clinical research. *Journal of the American Medical Informatics Association : JAMIA.* 2013;20(1):144-151.
31. Li P, Stuart EA, Allison DB. Multiple Imputation: A Flexible Tool for Handling Missing Data. *Jama.* 2015;314(18):1966-1967.
