# Supplementary material for: Rationale and design of the Novel Uses of adaptive Designs to Guide provider Engagement in Electronic Health Records (NUDGE-EHR) pragmatic adaptive randomized trial: a trial protocol
Source: Implement Sci. 2021 Jan 7;16:9. doi: 10.1186/s13012-020-01078-9 (PMC7792313; doi:10.1186/s13012-020-01078-9)

SUPPLEMENT

Rationale and design of the Novel Uses of adaptive Designs to Guide provider Engagement in Electronic Health Records (NUDGE-EHR) pragmatic adaptive randomized trial: A Trial Protocol

APPENDIX TABLES

eTable 1. Behavioral principles in electronic health record tools tested in regression model

	FACTORS OF INTERVENTIONS TO TEST (1=yes, 0=no)							
**Study Arm**	Choice architecture (timing of information)	Follow-up boostering	Cold-state priming	Simplification	Sign-off approval timing	Pre-commitment/ consistency	Framing: Guideline risks	Receipt of any EHR tool
**1: Order entry alert**	0	0	0	0	0	0	0	1
**2: Encounter opening alert**	1	0	0	0	0	0	0	1
**3: Order entry alert + follow-up booster message**	0	1	0	0	0	0	0	1
**4: Encounter opening alert + follow-up booster message**	1	1	0	0	0	0	0	1
**5: Order entry alert + pre-visit priming message**	0	0	1	0	0	0	0	1
**6: Encounter opening alert + pre-visit priming message**	1	0	1	0	0	0	0	1
**7: Simplified order entry alert**	0	0	0	1	0	0	0	1
**8: Simplified Encounter opening alert**	1	0	0	1	0	0	0	1
**9: Order entry alert + sign-off approval alert**	0	0	0	0	1	0	0	1
**10: Encounter opening alert + sign-off approval alert**	1	0	0	0	1	0	0	1
**11: Pre-commitment alert + Order entry alert**	0	0	0	0	0	1	0	1
**12: Pre-commitment alert + Encounter opening alert**	1	0	0	0	0	1	0	1
**13: Order entry alert with different risk framing**	0	0	0	0	0	0	1	1
**14: Encounter opening alert with different risk framing**	1	0	0	0	0	0	1	1
**15: Non-enhanced standard alert**	0	0	0	0	0	0	0	1
**16: Usual care**	0	0	0	0	0	0	0	0

APPENDIX FIGURES

eFigure 1. Provider tapering information and talking points embedded in enhanced alerts (2 pages)

Tapering information and patient talking points:

Benzodiazepine and sedative hypnotic ("Z-drug") de-prescribing

1) What are the risks of benzodiazepines and sedative hypnotic ("Z-drugs")?

- All guidelines consider these **high-risk** medications for older adults, including chronic use at low doses.
- Risks include a 30% higher risk of falls, reduced cognition, and impaired driving similar to driving drunk.

2) How many patients can actually be tapered off benzodiazepines and Z-drugs?

- Studies show that **>80% of patients can discontinue these drugs** – even in those using them for >10 years.
  - Minor side effects are expected within the first few days but will improve.
  - With appropriate tapering, serious side events can be completely avoided.
- In order to taper, some patients with severe anxiety or other behavioral health conditions may benefit from a consultation with a specialist.

3) How are benzodiazepines and Z-drugs tapered?

- Most patients can complete a full taper in approximately 12 weeks.
  - Guidelines suggest decreasing patients' dose by ~25% every 2 weeks, and then slowly at the end.
  - Some patients may need to be stabilized on half their original dose for several weeks before completely tapering.
- **Follow-up** is recommended during the tapering process.

4) What preset tapers/instructions are in the benzodiazepine and Z-drug SmartSet?

- **"Recommended" tapers section:**
  - Once and twice daily taper orders for the drug/strength (e.g., diazepam 10mg) that is currently on the patient's medication list or is being ordered now
  - Separate tapers for reducing from full dose to half dose (1st half) and half dose to no dose (2nd half)
- **"Alternative" tapers:**
  - Three times daily taper orders for the drug/strength on the medication list or is being ordered now
  - Other taper orders for that drug (e.g., other diazepam strengths)
- **Patient instructions for the after-visit summary:**
  - Tapering directions for each type of taper that includes a dose-reducing calendar *(example below)*
  - Managing insomnia
  - Managing anxiety
- **Orders for alternative medications or referrals**
- Notes about tapers:
  - All tapering orders have directions for pharmacy dispensing in the "Note to Pharmacy" box and a SIG for patients.
  - You may need to modify orders, such as pill quantity if patients are taking >1 pill per dose. If you modify the order, please modify the patient instructions.

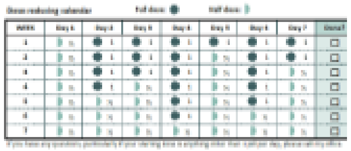

- Some formulations (e.g., capsules) cannot be split, so the SmartSet defaults to equivalent strengths of lorazepam for benzodiazepines and zolpidem for Z-drugs. You can also order manual tapers if needed.

5) What are the side effects that should be monitored?

- Monitor particularly for withdrawal symptoms, which may be a sign that the medication is being tapered too quickly.
  - Minor, short-term symptoms: rebound insomnia/anxiety, irritability, GI symptoms, dizziness
  - Severe symptoms: Confusion, delirium, seizures
- If symptoms are bothersome, maintain at the current dose for 1-2 weeks more before reducing further, as any reduction in use can be beneficial.

6) What are some patient talking points for de-prescribing these drugs?

Talking points: Risks of benzodiazepines/Z-drugs

- ***Highlight risks***
  - "Although these drugs can offer small short-term benefits, they can stop working and become dangerous. Serious side effects also remain over the long term, such as risk of falling or impairing your memory."
- ***Emphasize reasons to stop***
  - "To maintain your independence, we should remove any medications that increase the risks of serious side effects."

Talking points: Safe discontinuation/tapering

- ***Highlight tapering benefits***
  - "Many patients have successfully stopped taking this medication – the vast majority of people can stop taking them by safely reducing the dose slowly."
- ***Mention alternatives***
  - "There are other effective treatments than these medications for your [insomnia/anxiety]."

Talking points: Managing the tapering process

- ***Provide anticipatory guidance***
  - "You may experience a couple of nights of worse sleep."
  - "To reduce your risk of serious side effects, you might need to get through a few days of mild symptoms."
- ***Emphasize non-abrupt discontinuation***
  - "These medications should not be stopped immediately (unless directed by me or another provider)."

eFigure 2. SmartSet order set embedded within enhanced alerts

A. SmartSet as presented to providers

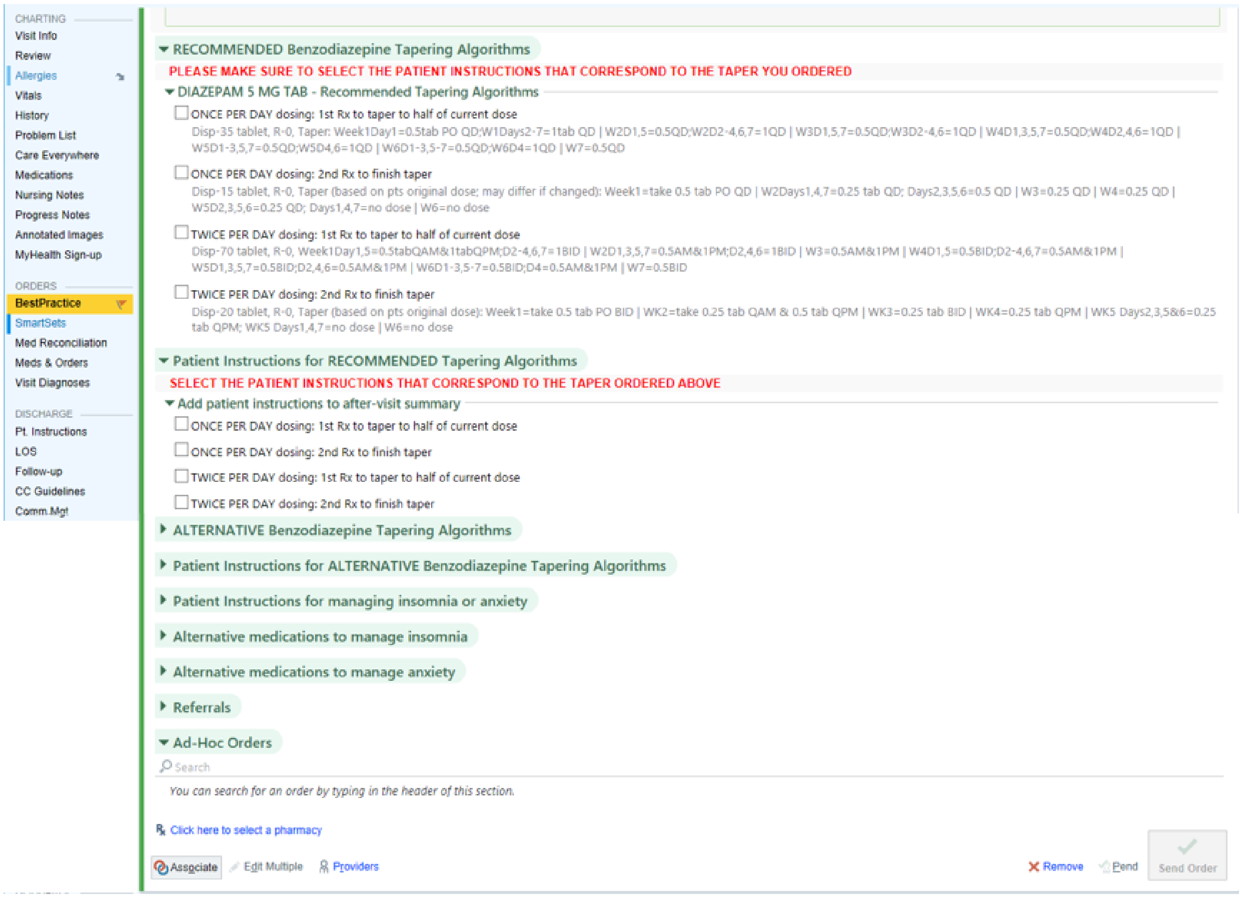

eFigure 3. Enhanced encounter opening alert used in Arms 2, 6, and 10

A. Part 1: Upon opening a face-to-face (in-person) encounter in a patient's chart

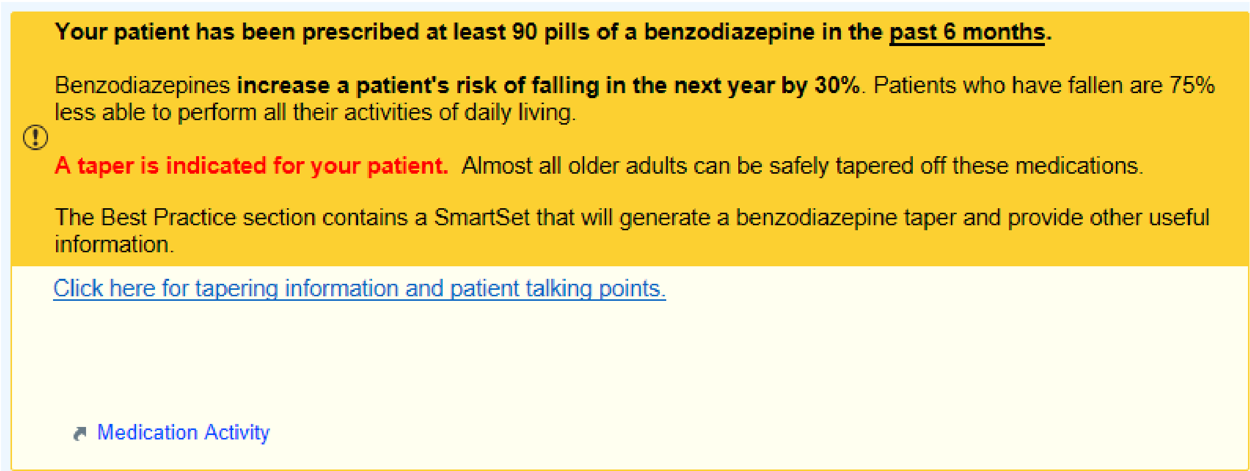

B. Part 2: Required to close the opened encounter in a patient's chart

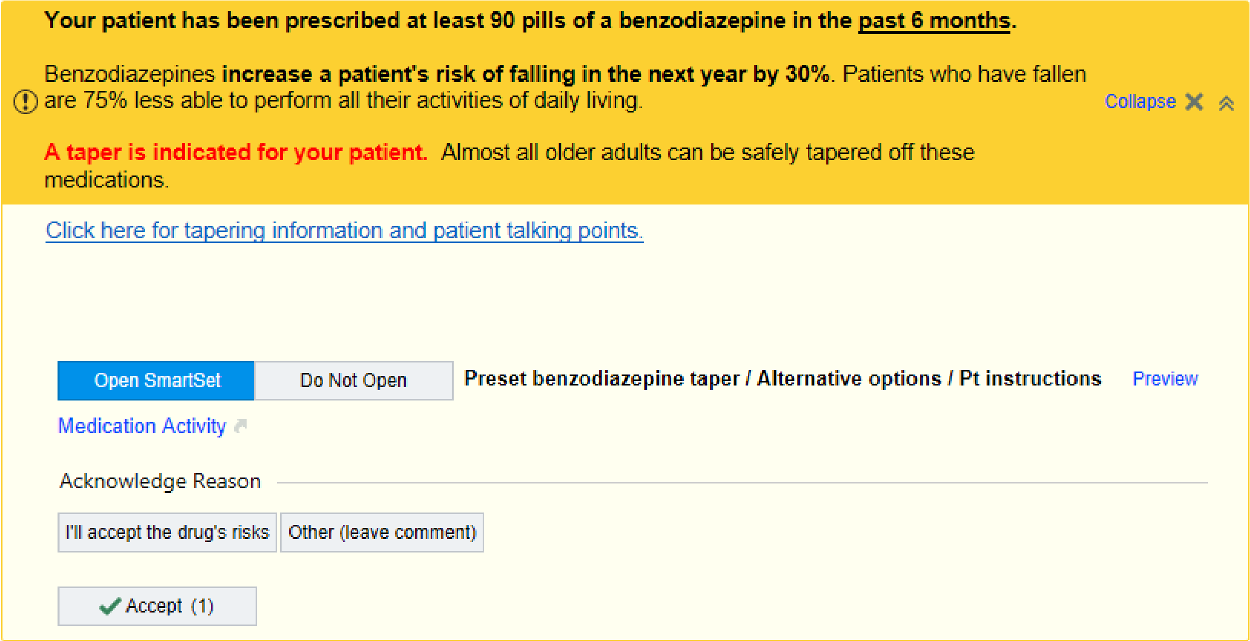

eFigure 4. Arm 3: Enhanced order entry alert + follow-up message

A. Order entry alert

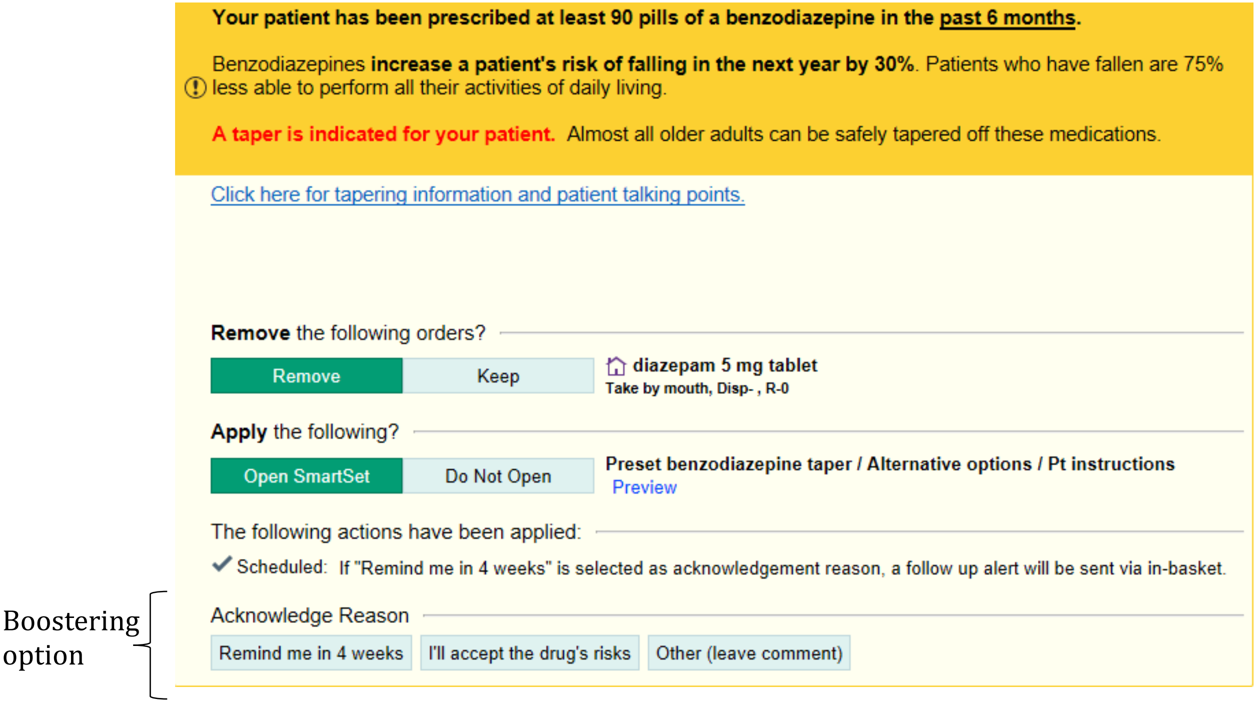

B. Follow-up message (4 weeks later) if "Remind me in 4 Weeks" was chosen

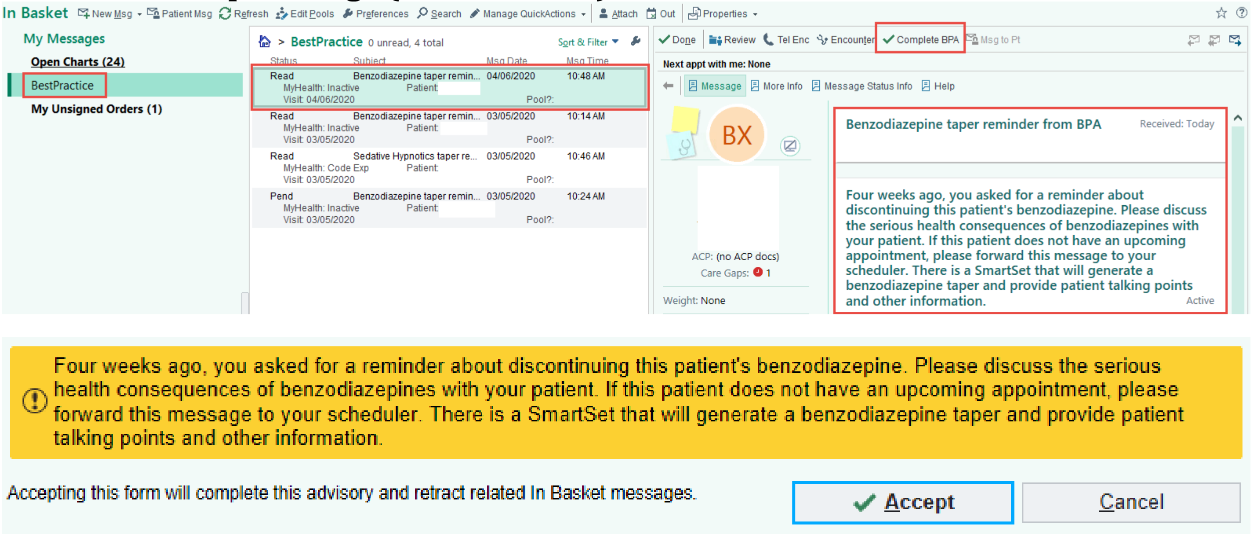

eFigure 5. Arm 7: Simplified enhanced order entry alert

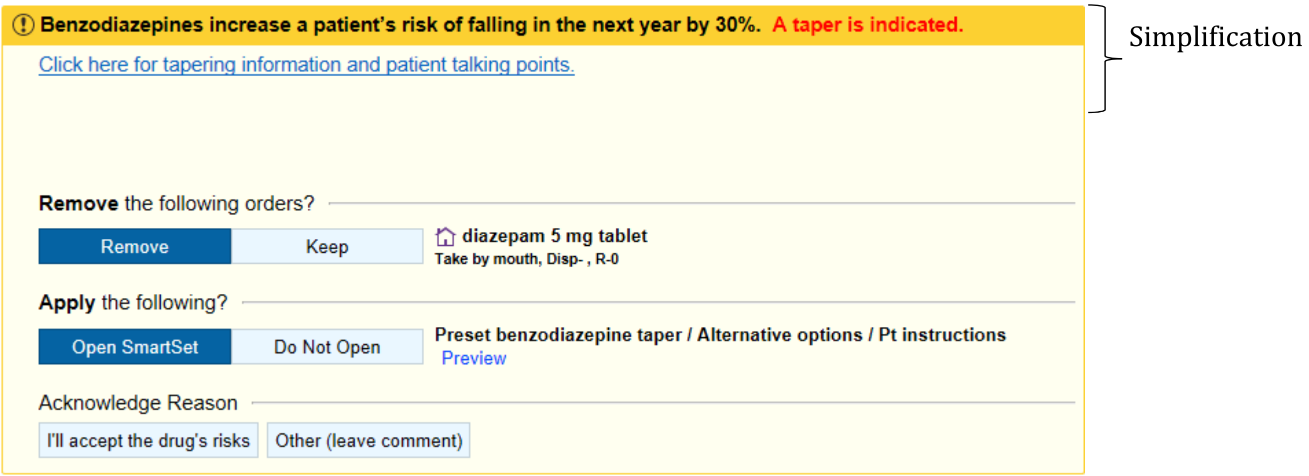

eFigure 6. Arms 9 and 10: Sign-off approval alert that triggers to providers when electronically signing off on refill medications ordered by support staff

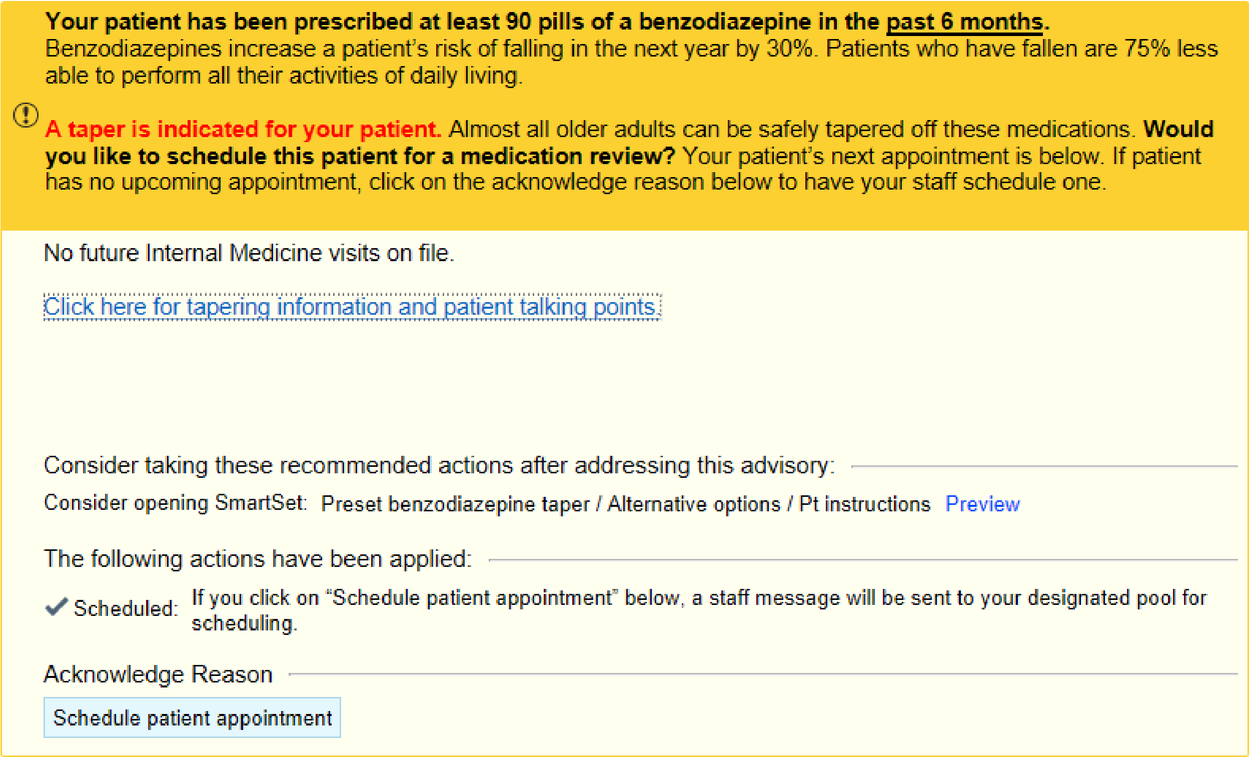

eFigure 7. Arm 11: Pre-commitment/consistency alert + enhanced order entry alert

A. Pre-commitment alert

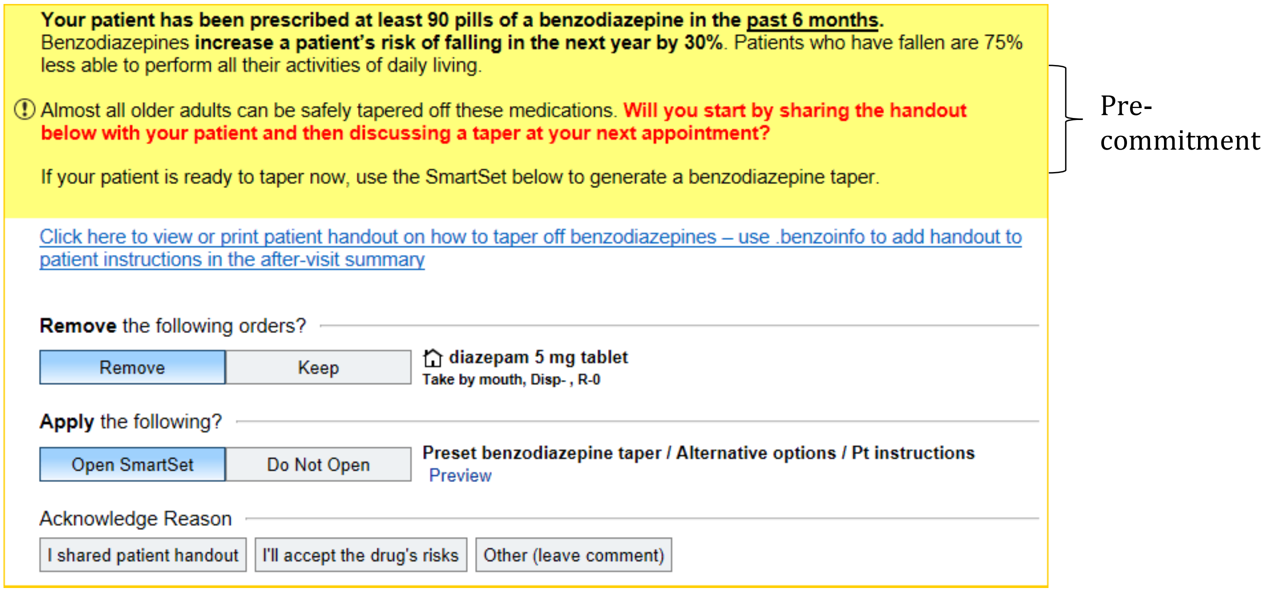

B. Enhanced order entry alert if "I shared patient handout" acknowledge reason chosen in pre-commitment alert

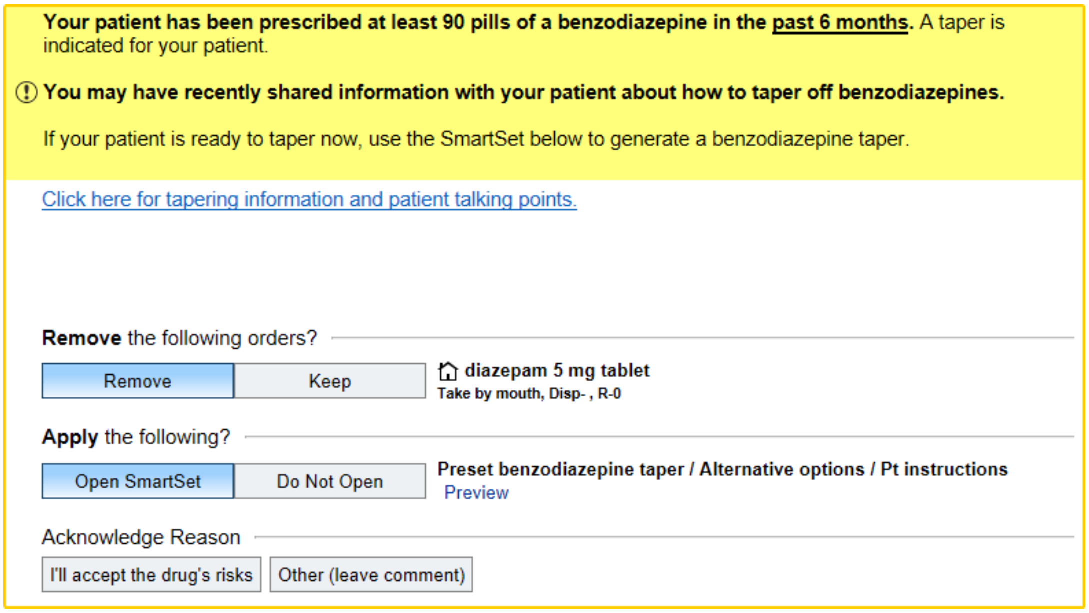

Note: If other acknowledge reasons are chosen, then the enhanced order entry alert shown in Figure 1 fires

eFigure 8. Arm 13: Enhanced order entry alert with different risk framing

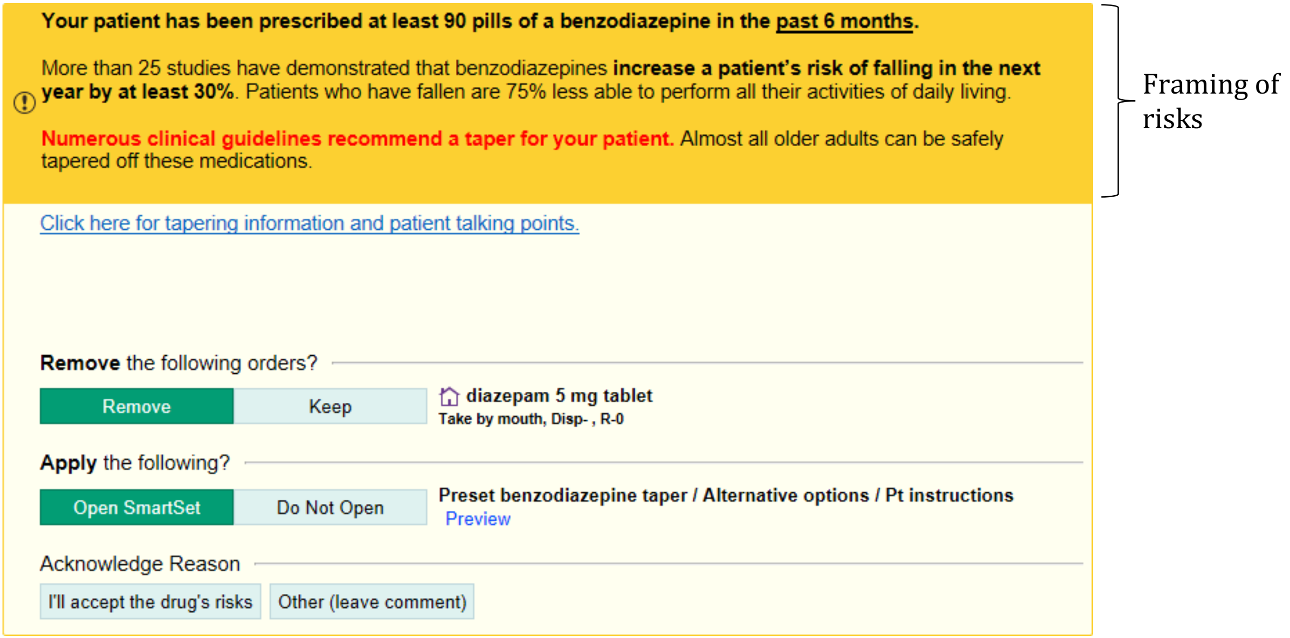

eFigure 9. Arm 15: Non-enhanced order entry alert

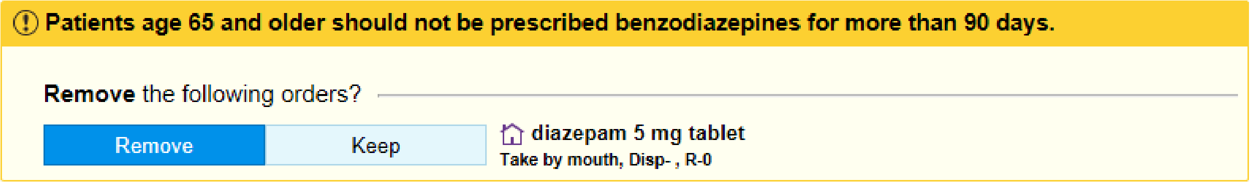

Supplement: Supplementary file 2 — Additional file 2: Supplement 2, eFigure 1 Provider tapering information and talking points embedded in enhanced alerts. Supplement 2, eFigure 2 SmartSet order set embedded within enhanced alerts. Supplement 2, eFigure 3 Enhanced encounter opening alert used in Arms 2, 6, and 10. Supplement 2, eFigure 4 Arm 3: Enhanced order entry alert + follow-up message. Supplement 2, eFigure 5 Arm 7: Simplified enhanced order entry alert. Supplement 2, eFigure 6 Arms 9 and 10: Sign-off approval alert that triggers to providers when electronically signing off on refill medications ordered by support staff. Supplement 2, eFigure 7 Arm 11: Pre-commitment/consistency alert + enhanced order entry alert. Supplement 2, eFigure 8 Arm 13: Enhanced order entry alert with different risk framing. Supplement 2, eFigure 9 Arm 15: Non-enhanced order entry alert. Supplement 2, eTable 1 Behavioral principles in electronic health record tools tested in regression model. [file 13012_2020_1078_MOESM2_ESM.pdf]
